# Ultra-fast optical ranging using quantum-dash mode-locked laser diodes

**DOI:** 10.1038/s41598-021-04368-4

**Published:** 2022-01-20

**Authors:** Philipp Trocha, Juned Nassir Kemal, Quentin Gaimard, Guy Aubin, François Lelarge, Abderrahim Ramdane, Wolfgang Freude, Sebastian Randel, Christian Koos

**Affiliations:** 1 Institute of Photonics and Quantum Electronics (IPQ), Karlsruhe Institute of Technology (KIT), Engesserstrasse 5, 76131 Karlsruhe, Germany; 2 Centre de Nanosciences et de Nanotechnologies CNRS, Université Paris-Saclay, Boulevard Thomas Gobert 10, 91220 Palaiseau, France; 3 Almae Technologies, Route de Nozay, 91460 Marcoussis, France; 4 Institute of Microstructure Technology (IMT), Karlsruhe Institute of Technology (KIT), Hermann-von-Helmholtz-Platz 1, 76344 Eggenstein-Leopoldshafen, Germany

**Keywords:** Integrated optics, Optoelectronic devices and components

## Abstract

Laser-based light detection and ranging (LiDAR) is key to many applications in science and industry. For many use cases, compactness and power efficiency are key, especially in high-volume applications such as industrial sensing, navigation of autonomous objects, or digitization of 3D scenes using hand-held devices. In this context, comb-based ranging systems are of particular interest, combining high accuracy with high measurement speed. However, the technical complexity of miniaturized comb sources is still prohibitive for many applications, in particular when high optical output powers and high efficiency are required. Here we show that quantum-dash mode-locked laser diodes (QD-MLLD) offer a particularly attractive route towards high-performance chip-scale ranging systems. QD-MLLDs are compact, can be easily operated by a simple DC drive current, and provide spectrally flat frequency combs with bandwidths in excess of 2 THz, thus lending themselves to coherent dual-comb ranging. In our experiments, we show measurement rates of up to 500 MHz—the highest rate demonstrated with any ranging system so far. We attain reliable measurement results with optical return powers of only – 40 dBm, corresponding to a total loss of 49 dB in the ranging path, which corresponds to the highest loss tolerance demonstrated so far for dual-comb ranging with chip-scale comb sources. Combing QD-MLLDs with advanced silicon photonic receivers offers an attractive route towards robust and technically simple chip-scale LiDAR systems.

## Introduction

Optical distance metrology is key to many applications in science and industry^1–5^. Among various techniques, dual-comb ranging based on multi-heterodyne detection^6–9^ stands out due to a unique combination of measurement accuracy and acquisition speed. As an example, dual-comb ranging based on mode-locked fiber lasers was demonstrated to provide a measurement precision of 5 nm, achieved by combining a time-of-flight scheme with optical interferometry^9^. More recently, soliton Kerr comb generators have gained importance as light sources for optical ranging^10,11^, permitting measurement rates of up to 100 MHz with sub-micrometer precision when used in dual-comb multi-heterodyne detection^7^. However, while these experiments demonstrate impressive performance parameters, the underlying comb sources are still rather complex, involving, e.g., discrete components such as fiber ring lasers^9^, high-power optical amplifiers^7^, or fiber-coupled electro-optic modulators ^6,8^. It might hence be challenging for these schemes to fulfil the stringent requirements with respect to robustness, size, weight and power consumption that are associated with many technically relevant applications. In addition, only few publications explicitly^6,12^ address the optical loss tolerance of comb-based ranging systems, which is a key performance metric in many ranging applications.

In this paper, we demonstrate that high-precision dual-comb ranging can be greatly simplified by using quantum-dash mode-locked laser diodes (QD-MLLD) as light sources. These devices are compact and robust and offer easy operation by a simple DC drive current^13,14^. QD-MLLDs provide spectrally flat frequency combs with line spacings of tens of gigahertz and have previously been used for high-speed optical communications^15–17^. In our experiments, we use a pair of QD-MLLDs with slightly detuned free spectral ranges (FSR) of approximately 50 GHz and demonstrate measurement rates of up to 500 MHz with a precision of 1.7 µm. To the best of our knowledge, this is the highest measurement rate demonstrated with any ranging system so far. When reducing the measurement rate to 10 kHz, the precision improves to 23 nm. We further demonstrate reliable ranging with optical return powers of only – 40 dBm, corresponding to a total round-trip loss of 49 dB in the free-space measurement path. To the best or our knowledge, this is the highest loss tolerance demonstrated so far for a comb-based measurement system that relies on chip-scale comb sources. When using an erbium-doped fiber amplifier (EDFA) for boosting the transmitted power, the maximum tolerable round-trip loss increases to 71 dB, without any impairment of the achievable precision. We demonstrate the measurement speed of our system by high-precision in-flight sampling of air-gun pellets moving at a speed of $150\;{\text{m}} \;{\text{s}}^{ - 1}$. We believe that our experiments pave the path towards practically viable chip-scale ranging systems that combine robust and technically simple frequency comb sources with advanced silicon photonic receivers^18^ and solid-state beam-steering circuits^5,19,20^, thus providing an unprecedented combination of compactness, accuracy, measurement speed, and loss tolerance.

### Comb-based integrated LiDAR systems and quantum-dash mode locked laser diodes

**Figure 1:**
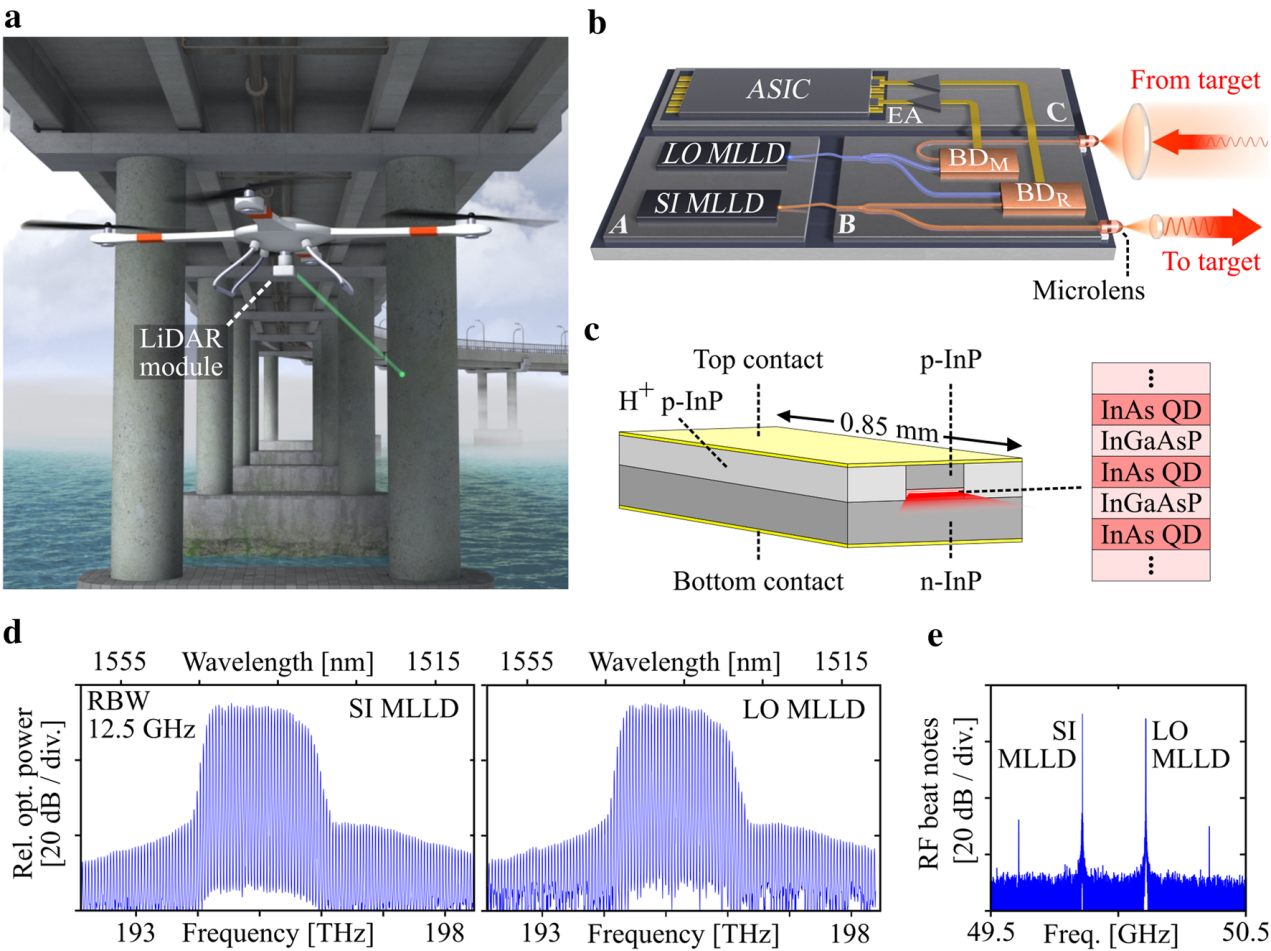
Application scenario and technical concept of an ultrafast chip-scale LiDAR system. (**a**) Compact lightweight LiDAR modules lend themselves to application in autonomously navigating carrier systems such as drones. High-resolution scanning of 3D surface profiles may complement 2D camera images for a reliable quantitative analysis of damage patterns, e.g., in structural health monitoring of large buildings or critical infrastructures. (**b**) Concept of a fully integrated dual-comb LiDAR system realized as a multi-chip assembly that combines a pair of MLLD-based frequency comb sources realized on InP substrates (A), silicon photonic transmitter and receiver circuits (B), and processing electronics (C) in a compact lightweight package. For distance measurements, light is emitted through a micro-lens directly attached to the chip, collimated by a macroscopic lens and directed towards the target via a beam scanner (not shown). The backscattered light is coupled back to the chip and sent to a balanced photodetector (BD_M_) for multi-heterodyne reception. An electronic application-specific integrated circuit (ASIC) is used for digital signal processing and extraction of the distance information. (**c**) Schematic of the QD-MLLD. The active medium comprises a stack of three InAs QD layers separated by InGaAsP layers. The pump current is applied via the bottom and top contact. The cavity is formed by the cleaved end facets of the chip with roughly 30% reflectivity each. Due to the inhomogeneously broadened gain spectrum of the QD material, multiple longitudinal lasing modes can oscillate simultaneously in the laser cavity, mode-locked by self-induced carrier density modulations. This leads to a time-periodic optical signal with a comb-like spectrum, where the FSR is determined by the roundtrip-time and hence the length of the cavity. (**d**) Left: Emission spectrum of the MLLD used as signal comb (SI MLLD), having an FSR of 49.72 GHz. Right: Emission spectrum of MLLD used as local oscillator (LO MLLD), for which the FSR amounts to 50.21 GHz. e RF beat notes of both MLLDs. The narrow RF linewidth of the order of 10 kHz indicates stable mode-locking of neighboring optical tones. The noise floor in this measurement is limited by the effective number of bits (ENOB ≈ 5) of the high-speed oscilloscopes (Keysight UXR0804A) that were used to digitize the signals, see Supplementary Information, Section ‘Noise impairments of recorded signals’ for a more detailed discussion.

An application scenario of an ultrafast chip-scale LiDAR system is illustrated in Fig. 1a. Due to its compact and lightweight implementation, the chip-scale LiDAR module can be mounted to autonomously navigating carrier systems such as drones, offering, e.g., new perspectives in structural health monitoring of large buildings or critical infrastructures such as bridges. In such applications, high-speed ranging is crucial for fast scanning of 3D surface profiles with sub-millimeter or even micrometer-scale precision during movement of the carrier system. The 3D surface profiles may complement 2D camera images for a reliable quantitative analysis of damage patterns. Figure 1b illustrates the concept of a fully integrated dual-comb LiDAR system that exploits multi-heterodyne reception for high-speed high-precision ranging^7,21^. The system is realized as a multi-chip assembly that combines a pair of MLLD-based frequency comb sources (A), silicon photonic transmitter and receiver circuits (B), as well as processing electronics (C) in a compact lightweight package. One MLLD emits the so-called signal frequency comb (SI MLLD, red) while the other one generates the local-oscillator (LO) comb (LO MLLD, blue). Photonic wire bonds^22,23^ are used to couple the frequency combs to the transmitter and receiver circuit, where they are split in two parts each. One part of the SI comb leaves the LiDAR system through a micro-lens directly attached to the chip^24^, is collimated by a macroscopic lens and directed towards the target. Note that the beam-scanning system is omitted in Fig. 1b for simplicity. The other part of the SI comb is superimposed with one part of the LO comb and received by a balanced reference photodetector (BD_R_). The LO MLLD and the SI MLLD are slightly detuned in center frequency and free-spectral range, leading to a photocurrent with comb-like spectrum that reveals the phase relations of the various optical tones^7,21^. The light backscattered from the target is collected by a large lens, focused into a waveguide of the receiver chip via a micro-lens^24^, and guided to a balanced measurement photodetector (BD_M_), where it is superimposed with the second portion of the LO comb. The distance can be extracted by comparing the phases of the spectral components in the comb-like photocurrent of the measurement detector to the phases extracted from the photocurrent of the reference detector. In the concept illustrated in Fig. 1b, the electrical output signals from BD_R_ and BD_M_ are transferred to chip (C) by electrical wire bonds, amplified, and processed by an application specific integrated circuit (ASIC). The data evaluation scheme exploits the concept of synthetic-wavelength interferometry and is described in more detail in Refs.^7,12^.

The MLLDs rely on InAs/InGaAsP quantum-dash (QD) structures on InP substrates and are driven by a simple DC pump current, see Fig. 1c. The active medium consists of three stacked layers of InAs QD, which are separated by InGaAsP barriers, such that charge carriers injected into the layer stack become trapped in the QD-layers and recombine radiatively. The optical field is vertically and laterally confined to the InAs-QD/InGaAsP stack through the surrounding low-index InP material. The cavity is formed by the cleaved end facets with roughly 30% reflectivity each. Multiple longitudinal lasing modes can oscillate simultaneously in this Fabry-Pérot laser cavity. These longitudinal modes experience mode-locking due to self-induced carrier density modulations of the gain medium^13^, leading to a time-periodic optical signal with a comb-like spectrum, where the FSR of the comb lines is determined by the roundtrip-time and hence the length of the cavity. The comb spectrum is centered around a photon energy of approximately 0.81 eV (195 THz, 1538 nm), with a relatively large optical bandwidth of more than 1.5 … 2 THz, owing to the inhomogeneously broadened gain spectrum arising from the shape and size variations of the quantum dashes.

In the following, we denote the comb-line frequencies by $\omega_{{{\text{SI,}}\mu }}$ for the SI MLLD and by $\omega_{{{\text{LO,}}\mu^{\prime}}}$ for the LO MLLD,

1 $$\omega_{{{\text{SI}},\mu }} = \omega_{{{\text{SI}},0}} + \mu \,\omega_{{\text{SI,r}}} ,$$

2 $$\omega_{{{\text{LO}},\mu^{\prime}}} = \omega_{{{\text{LO}},0}} + \mu^{\prime}\,\omega_{{\text{LO,r}}} .$$

In these relations, $\omega_{{{\text{SI,}}0}}$ and $\omega_{{{\text{LO,}}0}}$ are the center comb lines, the integer comb line indices are denoted as $\mu$ for the SI comb and as $\mu^{\prime}$ for the LO comb, and the corresponding free spectral ranges are $\omega_{{\text{SI,r}}}$ and $\omega_{{\text{LO,r}}}$, respectively. Spectra of the SI-MLLD comb and the LO-MLLD comb are shown in Fig. 1d. To determine the FSR of the combs experimentally, we connect both combs simultaneously to a photodetector with a 3 dB-bandwidth of 43 GHz, record the photocurrent and compute the Fourier transform. Due to the limited photodetector bandwidth, only mixing products of directly neighboring comb lines of each source are visible in the RF beat signal, see Fig. 1e. The left beat signal corresponds to the FSR $\omega_{{\text{SI,r}}} = 2\pi \times 49.72\;{\text{GHz}}$ of the SI MLLD, and the right beat signal reveals the FSR $\omega_{{\text{LO,r}}} = 2\pi \times 50.21\;{\text{GHz}}$ of the LO MLLD. The 3 dB-bandwidth of the individual beat notes, also referred to as the RF linewidth of the QD MLLD comb, is typically of the order of 10 kHz^15^, which indicates efficient mode-locking of the longitudinal modes. Note that the RF linewidth only indicates the low relative phase noise of neighboring comb tones. The absolute phase noise of the tones, as, e.g., measured with respect to an ultra-stable continuous-wave (cw) reference tone, is usually much stronger, indicated by optical linewidths in the order of 10 MHz^15^ or more, see Section ‘System, operation principle and digital signal processing’ for details. In contrast to other comb generators, QD-MLLDs emit frequency combs by simply applying a DC current^13,14^, without the need for high-speed RF devices^6,8,21^ or fiber-based amplifiers^7,9^. Typically, QD-MLLDs offer around 50 comb lines with FSR in the range of 10 GHz to 100 GHz. In our devices, the total comb power amounts to 20 mW with an average line power of 400 µW. The simple operation of QD-MLLDs and their relatively large power per comb line along with the potential for hybrid or monolithic integration with photonic integrated circuits make the devices attractive for chip-scale LiDAR systems.

## Methods

### System, operation principle and digital signal processing

**Figure 2:**
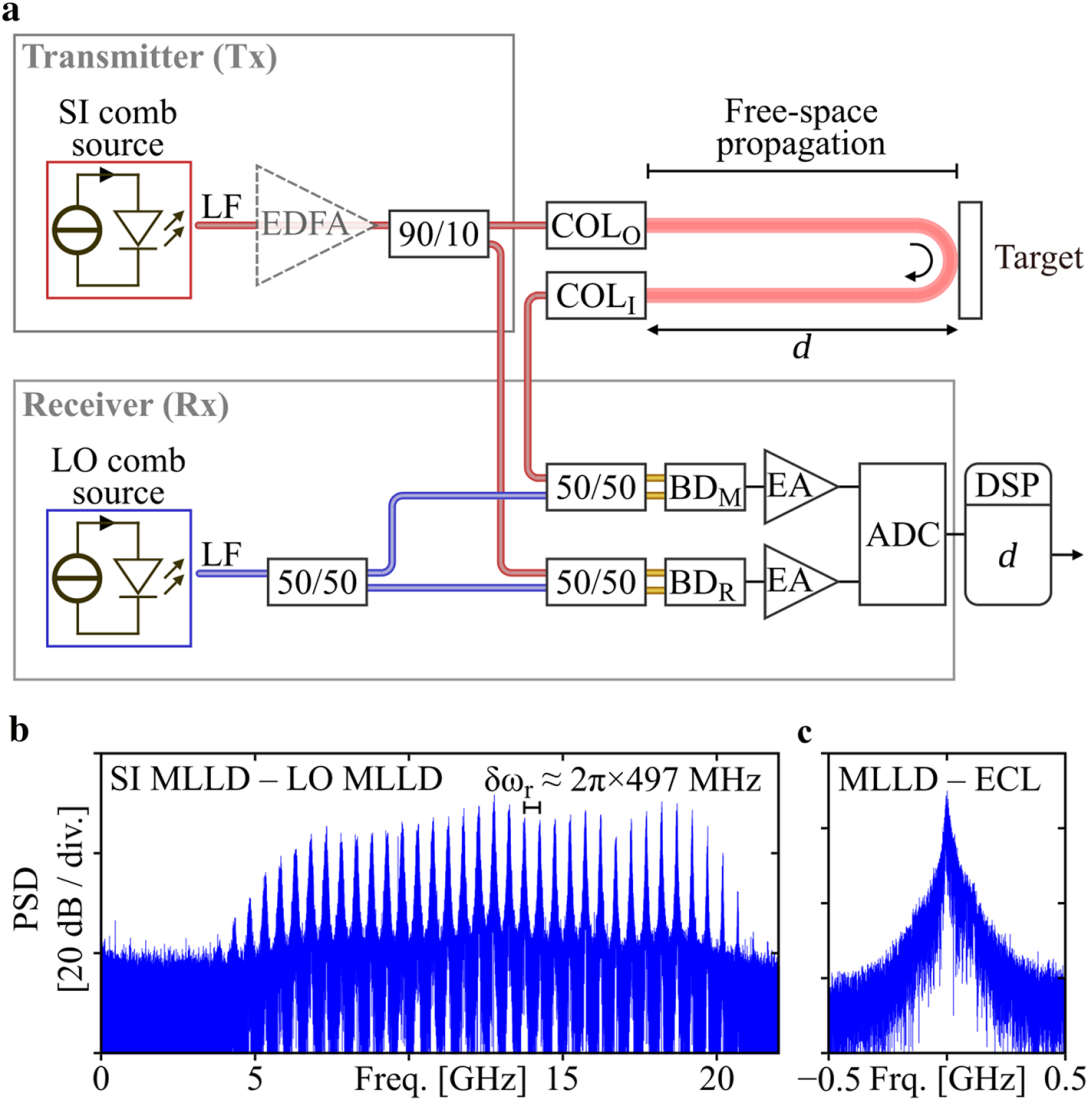
Experimental setup and measured RF spectra of our proof-of-concept demonstration. (**a**) Experimental setup: In the transmitter (Tx), the signal (SI) comb generated by a first MLLD is coupled into a lensed fiber (LF), optionally amplified in an EDFA (dashed lines), and then split by a 90/10 coupler. A first portion is emitted towards the target through an output collimator (COL_O_). After free-space propagation and reflection at the target, the comb re-enters the system at the input collimator (COL_I_). In the receiver (Rx), the captured light is sent to a 50/50 coupler where it is superimposed with a first portion of the local oscillator (LO) comb emitted by a second MLLD. The superimposed signals are then sent to a balanced photodetector, which is referred as the measurement detector (BD_M_). An electrical amplifier (EA) boosts the electrical signal, which is captured by a high-speed analog-to-digital converter (ADC). The second portion of the SI comb is superimposed with a second portion of the LO comb in another 50/50 coupler, detected by a reference photodetector (BD_R_), and fed to a second channel of the ADC. Digital signal processing (DSP) of the recorded signals is performed offline to obtain the distance *d* from the recorded signals. Details regarding the components used in the experimental setup can be found in the Supplementary Information, Section ‘Detailed description of the experimental setup’. (**b**) Power spectral density (PSD) of the electric signal emitted, as obtained from the measurement photodetector for an incident signal power of − 5 dBm. The spectrum shows discrete RF tones that are generated by mixing of pairs of optical tones of the SI MLLD and the LO MLLD. The comb line spacing is approximately 497 MHz, corresponding to the FSR difference of the two comb sources. The RF tones exhibit substantial linewidths of 10 MHz and more, which vary across the spectrum and which are caused by the rather high optical linewidth of the corresponding comb lines. Also here, the noise floor is limited by the effective number of bits (ENOB ≈ 5) of the high-speed oscilloscopes (Keysight UXR0804A) that were used to digitize the signals, see Supplementary Information, Section ‘Noise impairments of recorded signals’ for a more detailed discussion. (**c**) Beat note of a QD-MLLD comb line with a narrow-linewidth ECL, showing an optical 3-dB linewidth of approximately 15 MHz.

In our proof-of-principle experiments we use the setup depicted in Fig. 2a, which consists of a transmitter (Tx) and a receiver (Rx) part. The SI MLLD at the transmitter is driven by a DC pump current of 200 mA at a voltage of 1.8 V, leading to a single-facet optical output power of 13 dBm. This corresponds to a wall-plug efficiency (WPE) of 5.6%, which compares well to that of continuous-wave laser diodes. The output of the SI MLLD is coupled into a lensed fiber (LF) with a coupling loss of approximately $1\;{\text{dB}}$. A fiber-optic circulator (not shown) attached to the lensed fiber prevents spurious back-reflections into the MLLD. Optionally, the signal comb power can be amplified using an erbium-doped fiber amplifier (EDFA, dashed). The LO comb is generated in an equivalent setup, which does not contain an amplifier. We split the SI comb in two parts using a 90/10 coupler. Light leaving the 90%-port is collimated (COL_O_) and radiated towards the target located at a distance *d*. The emitted optical power amounts to 9 dBm without and to 31 dBm with EDFA. The collimated signal beam is scattered back from the target and collected by a second collimator (COL_I_) after traversing the measurement path with a free-space length of $2d$. A more detailed description of the free-space optics is given in the Supplementary Information, Section ‘Free-space optical setup for compensation of fiber drift’. In the receiver, the LO comb is split by a 50/50 coupler. Signal light returning from the measurement path is superimposed with light of the LO comb in another 50/50 coupler and received with a balanced photodetector (BD_M_). The second fraction of the SI comb does not leave the setup and is directly superimposed with the second fraction of the LO comb in another 50/50 coupler and sent to a balanced reference photodetector (BD_R_). The electrical signals are amplified by electrical amplifiers (EA) and recorded using a high-speed oscilloscope (Keysight UXR0804A) with an analog-to-digital converter (ADC) operated at a sampling rate of $128\;{\text{GSa}} \;{\text{s}}^{ - 1}$, such that the RF beat notes of the individual combs at approximately 50 GHz can be extracted for exact determination of the respective FSR, see Fig. 1e. More detailed information regarding the components used in the experimental setup can be found in the Supplementary Information, Section ‘Detailed description of the experimental setup’. Digital signal processing (DSP) is performed offline to extract the target distance $d$. Figure 2b shows the power spectral density (PSD) of the RF beat signal between the SI-MLLD and the LO-MLLD as extracted from BD_M_. In general, the PSD contains all RF beat notes of the SI comb lines, Eq. (1), (index $\mu$) and the LO comb lines, Eq. (2), (index $\mu^{\prime}$), appearing at RF frequencies^7^

3 $$\omega_{{\mu^{\prime},\mu }} = \left| {\omega_{{{\text{LO}},0}} - \omega_{{{\text{SI}},0}} + \left( {\mu^{\prime} - \mu } \right)\omega_{{\text{SI,r}}} + \mu^{\prime}\left| {\omega_{{\text{LO,r}}} - \omega_{{\text{SI,r}}} } \right|} \right|.$$

After low-pass filtering of the RF signal, only beat notes of LO comb lines with the respective nearest SI comb lines are retained, which are represented by $\mu^{\prime} = \mu$ in Eq. (3), such that $\omega_{\mu } = \left| {\omega_{{{\text{LO}},0}} - \omega_{{{\text{SI}},0}} + \mu \left| {\omega_{{\text{LO,r}}} - \omega_{{\text{SI,r}}} } \right|} \right|$, see Fig. 2b. In our measurement, the center frequency $\left| {\omega_{{{\text{LO}},0}} - \omega_{{{\text{SI}},0}} } \right| = \omega_{0}$ is located at $2\pi \times 12\;{\text{GHz}}$ and is adjusted by temperature tuning of the MLLDs via small pump current changes such that the mixing products of the two combs can be clearly separated. The spectral separation $\left| {\omega_{{\text{LO,r}}} - \omega_{{\text{SI,r}}} } \right| = \delta \omega_{{\text{r}}}$ of the beat signals is approximately about $2\pi \times 497\;{\text{MHz}}$. This FSR difference may drift by a few MHz on a long time-scale, unless stabilization techniques are used^25^.

For extracting the distance, we evaluate the phases $\Phi_{{{\text{meas,}}\mu }}$ of the RF beat notes at the output of BD_M_ and compare them to the corresponding phases $\Phi_{{{\text{ref,}}\mu }}$ extracted from BD_R_. The phases $\Phi_{{{\text{meas,}}\mu }} \left( d \right)$ depend on the target distance $d$, whereas the phases $\Phi_{{{\text{ref,}}\mu }}$ are independent of $d$ and serve as a reference for the initial phases of the various tones, see Ref.^7^ for a detailed mathematical description of the measurement technique. The free-space distance $d$ can be determined from the phases differences $\delta \Phi_{\mu } (d) = \Phi_{{{\text{meas}},\mu }} (d) - \Phi_{{{\text{ref}},\mu }}$ of the various RF beat notes^7^,

4 $$\delta \Phi_{\mu } (d) = \mu \times \left( {{{\omega_{{\text{SI,r}}} } \mathord{\left/ {\vphantom {{\omega_{{\text{SI,r}}} } {c_{0} }}} \right. \kern-\nulldelimiterspace} {c_{0} }}} \right)n_{{{\text{air}}}} \times 2\left( {d - d_{0} } \right),\quad n_{{{\text{air}}}} = 1.000226.$$

In this relation, $c_{0}$ denotes the vacuum speed of light, and the refractive index of air $n_{{{\text{air}}}}$ was obtained from Ciddor’s formula^26^. The quantity $d_{0}$ denotes a constant distance offset dictated by the lengths of the various fibers between the comb sources and the balanced detectors, see Ref.^7^ for details.

Note that the RF beat notes shown in Fig. 2b exhibit substantial linewidths of 10 MHz and more, which is caused by the rather high optical linewidth of the individual comb lines. For comparison, we also record a beat note of an MLLD comb line with a highly stable tunable external-cavity laser (ECL, linewidth < 10 kHz), see Fig. 2c, which exhibits an optical linewidth of approximately 15 MHz. These numbers are typical for QD-MLLD, see Ref.^16^ for a more detailed discussion. For the measurements presented here, phase noise does not have a significant impact on the result since the path differences are much smaller than the coherence length ${{c_{0} } \mathord{\left/ {\vphantom {{c_{0} } {15\;{\text{MHz}} }}} \right. \kern-\nulldelimiterspace} {15\;{\text{MHz}} }} = 20\;{\text{m}}$. The phase noise of the beat notes on the reference and the measurement detector is hence strongly correlated and does not have strong impact on the phase differences $\delta \Phi_{\mu } (d) = \Phi_{{{\text{meas}},\mu }} (d) - \Phi_{{{\text{ref}},\mu }}$. For larger measurement distances, it is possible to additionally apply linewidth-reduction techniques for the MLLDs, exploiting, e.g., external-cavity feedback^17,27,28^ or injection locking^14^.

For extracting the distance information from the measured phases, we numerically unwrap the measured phases by adding integer multiples of $2\pi$ to each phase difference $\delta \Phi_{\mu } (d)$ such that the pairs $\left( {\mu ,\delta \Phi_{\mu } (d)} \right)$ can be fitted by a straight line according to Eq. (4). From the slope of this fit, we then determine $d$. Note that the $2\pi$-ambiguity of the phase differences $\delta \Phi_{\mu } (d)$ leads to an ambiguity of the slope and hence to an unambiguity distance $d_{{{\text{ua}}}} = {{2\pi c_{0} } \mathord{\left/ {\vphantom {{2\pi c_{0} } {\left( {2n_{{{\text{air}}}} \omega_{{\text{SI,r}}} } \right)}}} \right. \kern-\nulldelimiterspace} {\left( {2n_{{{\text{air}}}} \omega_{{\text{SI,r}}} } \right)}} = 3.01\;{\text{mm}}$ of the measured distance *d*. The minimum observation time needed for evaluating the phases $\Phi_{{{\text{meas}},\mu }} (d)$ and $\Phi_{{{\text{ref}},\mu }}$ is the period ${{T_{{\text{r}}} = 2\pi } \mathord{\left/ {\vphantom {{T_{{\text{r}}} = 2\pi } {\delta \omega_{{\text{r}}} }}} \right. \kern-\nulldelimiterspace} {\delta \omega_{{\text{r}}} }} = 2.02\;{\text{ns}}$ of the SI-MLLD–LO-MLLD beat signal generated on the two balanced photodetectors. For smaller evaluation times $T_{{{\text{eval}}}}$, the frequency resolution $T_{{{\text{eval}}}}^{ - 1}$ would be insufficient to discriminate neighboring RF beat notes at frequencies $\omega_{\mu }$. Figure 2b shows the calculated power spectrum of a sequence of $N_{{{\text{eval}}}} = 4000$ pulse periods $T_{{\text{r}}}$ at a received signal comb power of − 5 dBm. The evaluation time $T_{{{\text{eval}}}} = N_{{{\text{eval}}}} T_{{\text{r}}}$ and the corresponding frequency resolution $T_{{{\text{eval}}}}^{ - 1}$ define the effective noise-filtering bandwidth, which we call evaluation bandwidth and which, in case of Fig. 2b, amounts to $B_{{{\text{eval}}}} = {1 \mathord{\left/ {\vphantom {1 {\left( {N_{{{\text{eval}}}} \,T_{{\text{r}}} } \right)}}} \right. \kern-\nulldelimiterspace} {\left( {N_{{{\text{eval}}}} \,T_{{\text{r}}} } \right)}} = 124\;{\text{kHz}}$. While this narrow noise-filtering bandwidth $B_{{{\text{eval}}}}$ suppresses noise effectively and therefore results in an accurate spectrum, it is not the setting of choice if evaluation speed is important. For high-speed measurements, we may choose $N_{{{\text{eval}}}} = 1$, i.e., $B_{{{\text{eval}}}} = 497\;{\text{MHz}}$, which can be increased to $N_{{{\text{eval}}}} = 10$, i.e., $B_{{\text{eval}}} = 49.7\;{\text{MHz}}$ in case an increased signal-to-noise power ratio is needed.

The measured phase differences $\delta \Phi_{\mu } (d)$ of the $N_{{\text{b}}}$ beat notes are subject to various impairments such as shot noise, electronic noise of the receiver circuits, or impairments of the ADC, which makes the extracted distances *d* unreliable, see Sections ‘Noise impairments of recorded signals’ and ‘Impact of shot noise on the measurement precision’ of the Supplementary Information for details. As a reliability metric for each measured distance, we extract the residual errors of the data points $\left( {\mu ,\delta \Phi_{\mu } (d)} \right)$ with respect to the linear fit. We define an overall fit error $\varepsilon$ as the root-mean-square of the fit errors of the $N_{{\text{b}}} \approx 25$ fitted beat-note phases

5 $$\varepsilon \left( {d_{i} } \right) = \sqrt {\frac{1}{{N_{{\text{b}}} - 1}}\sum\limits_{{\mu = \left\lfloor { - N_{{\text{b}}} /2} \right\rfloor + 1}}^{{\left\lfloor {N_{{\text{b}}} /2} \right\rfloor }} {\left( {\delta \Phi_{{\mu ,{\text{meas}}}} (d_{i} ) - \delta \Phi_{{\mu ,{\text{fit}}}} \left( {d_{i} } \right)} \right)^{2} } } ,$$

where the floor operator $\left\lfloor \cdot \right\rfloor$ denotes the nearest smaller integer. If $\varepsilon (d_{i} )$ is small, the linear fit is a good approximation to the measured phase differences $\delta \Phi_{\mu } (d)$, and the result should be reliable. In contrast, if $\varepsilon (d_{i} )$ is high, impairment due to noise may be substantial. We define a limit $\varepsilon_{{{\text{th}}}} (d_{i} )$ to distinguish between reliable distance data points, where $\varepsilon (d_{i} ) < \varepsilon_{{{\text{th}}}} (d_{i} )$, and unreliable distance data points defined by $\varepsilon (d_{i} ) \ge \varepsilon_{{{\text{th}}}} (d_{i} )$, which are eventually discarded. For details on the determination of the fit error threshold $\varepsilon_{{{\text{th}}}} (d_{i} )$, see the Supplementary Information, Section ‘Selection of reliable distance data by fit error’.

## Results

### System precision and accuracy

In our experiments, we characterize the achievable precision of the system by repeatedly measuring the distance to a fixed target mirror. We record time series of an overall duration of 1.56 ms, limited by the memory size of the oscilloscope. For finding the distance, we evaluate the same recording for two different evaluation times $T_{{{\text{eval}}}} = N_{{{\text{eval}}}} T_{{\text{r}}}$ with $N_{{{\text{eval}}}} = 1$ ($B_{{{\text{eval}}}} = 495\;{\text{MHz}}$), and $N_{{{\text{eval}}}} = 10$ ($B_{{\text{eval}}} = 49.5\;{\text{MHz}}$). We extract $N_{{\text{d}}}$ distance values, $10^{5} \le N_{{\text{d}}} \le 10^{6}$, and compute the Allan deviation $\sigma_{A}$ as a function of the averaging time $\tau$. To this end introduce the number of averaged distance values

6 $$N_{{{\text{av}}}} = \left\lfloor {\tau B_{{{\text{eval}}}} } \right\rfloor = \left\lfloor {\frac{\tau }{{N_{{{\text{eval}}}} T_{{\text{r}}} }}} \right\rfloor .$$

The number $N_{{{\text{av}}}}$ of averaged samples leads to the averaged distance values $\overline{d}_{j} \left( {N_{{{\text{av}}}} } \right)$,

7 $$\overline{d}_{j} \left( {N_{{{\text{av}}}} } \right) = \frac{1}{{N_{{{\text{av}}}} }}\mathop \sum \limits_{i = 0}^{{N_{{{\text{av}}}} - 1}} d_{{j{\mkern 1mu} N_{{{\text{av}}}} + i}} ,$$

based on which we calculate the Allan deviation^29^,

8 $$\sigma_{A}^{2} \left( {N_{{{\text{av}}}} } \right) = \frac{1}{2}\frac{1}{{\left\lfloor {{{N_{{\text{d}}} } \mathord{\left/ {\vphantom {{N_{{\text{d}}} } {N_{{{\text{av}}}} }}} \right. \kern-\nulldelimiterspace} {N_{{{\text{av}}}} }}} \right\rfloor - 1}}\sum\limits_{j = 1}^{{\left\lfloor {{{N_{{\text{d}}} } \mathord{\left/ {\vphantom {{N_{{\text{d}}} } {N_{{{\text{av}}}} }}} \right. \kern-\nulldelimiterspace} {N_{{{\text{av}}}} }}} \right\rfloor {\kern 1pt} - 1}} {\left( {\overline{d}_{j + 1} \left( {N_{{{\text{av}}}} } \right) - \overline{d}_{j} \left( {N_{{{\text{av}}}} } \right)} \right)^{2} } .$$

For comparison, we also compute the standard deviation $\sigma \left( {N_{{{\text{av}}}} } \right)$ of all measured distances to the fixed target mirror as a function of the number of averaged samples,

9 $$\begin{aligned} \sigma_{{\overline{d}}}^{2} \left( {N_{{{\text{av}}}} } \right) & = \frac{1}{{\left\lfloor {{{N_{{\text{d}}} } \mathord{\left/ {\vphantom {{N_{{\text{d}}} } {N_{{{\text{av}}}} }}} \right. \kern-\nulldelimiterspace} {N_{{{\text{av}}}} }}} \right\rfloor - 1}}\sum\limits_{j = 1}^{{\left\lfloor {{{N_{{\text{d}}} } \mathord{\left/ {\vphantom {{N_{{\text{d}}} } {N_{{{\text{av}}}} }}} \right. \kern-\nulldelimiterspace} {N_{{{\text{av}}}} }}} \right\rfloor {\kern 1pt} }} {\left( {\overline{d}_{j} \left( {N_{{{\text{av}}}} } \right) - \overline{{\overline{d}_{j} \left( {N_{{{\text{av}}}} } \right)}} } \right)^{2} } , \\ \overline{{\overline{d}_{j} \left( {N_{{{\text{av}}}} } \right)}} & = \frac{1}{{\left\lfloor {{{N_{{\text{d}}} } \mathord{\left/ {\vphantom {{N_{{\text{d}}} } {N_{{{\text{av}}}} }}} \right. \kern-\nulldelimiterspace} {N_{{{\text{av}}}} }}} \right\rfloor }}\sum\limits_{j = 1}^{{\left\lfloor {{{N_{{\text{d}}} } \mathord{\left/ {\vphantom {{N_{{\text{d}}} } {N_{{{\text{av}}}} }}} \right. \kern-\nulldelimiterspace} {N_{{{\text{av}}}} }}} \right\rfloor }} {\overline{d}_{j} \left( {N_{{{\text{av}}}} } \right)} . \\ \end{aligned}$$

Note that distance points $d_{i}$ for which the fit error $\varepsilon (d_{i} )$ exceeds the threshold $\varepsilon_{{{\text{th}}}} (d_{i} )$ are not considered when evaluating Eqs. (6)…(9).

**Figure 3:**
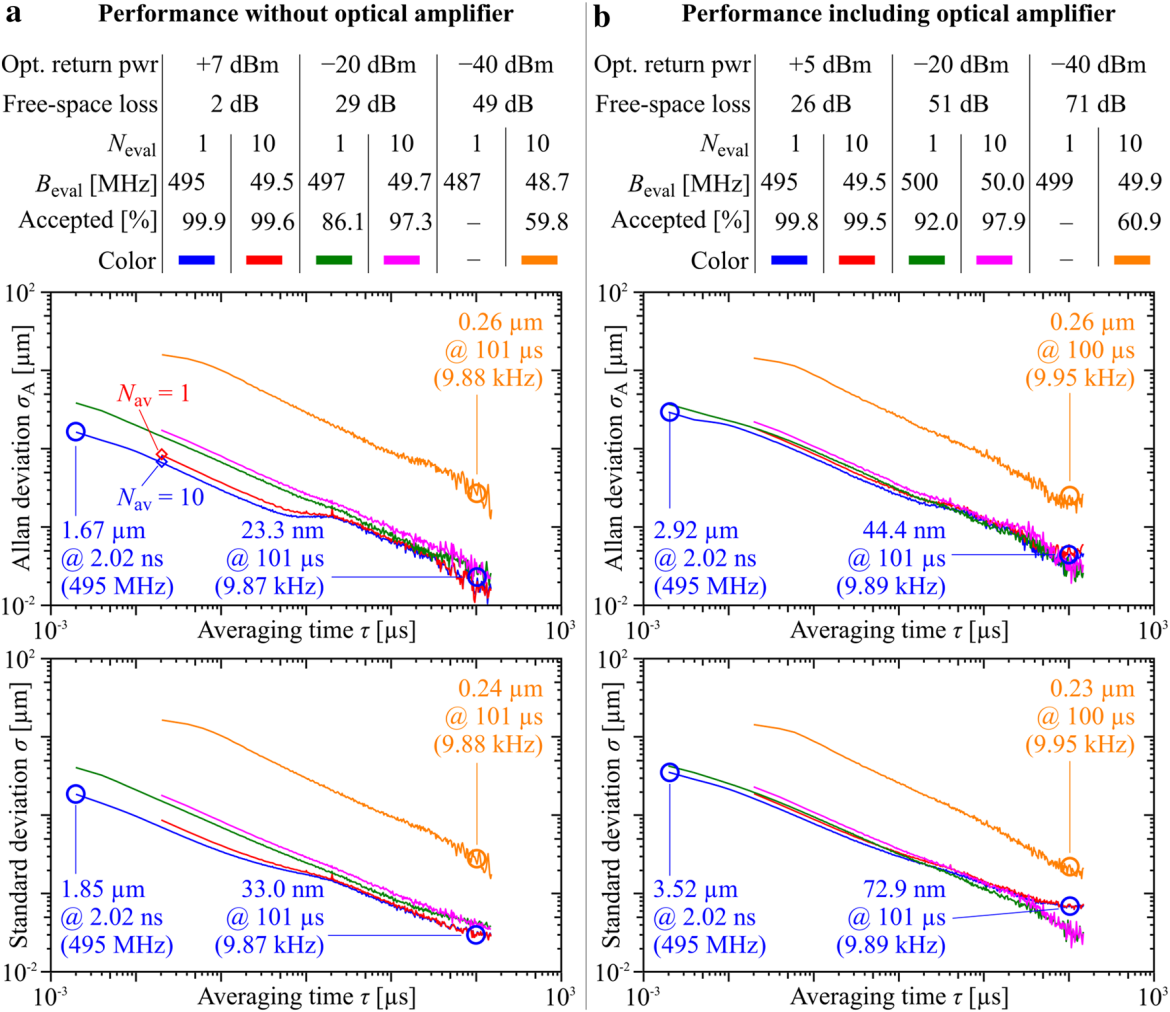
Performance of ranging system for varying optical return powers, with and without booster optical amplifier (EDFA), see Fig. 2a for the underlying experimental setup and Section ‘Detailed description of experimental setups’ of the Supplementary Information for further details. (**a**) Performance without EDFA. The table lists the optical return power, the associated round-trip loss in the free-space path, the number $N_{{{\text{eval}}}}$ of repetition periods $T_{{\text{r}}}$ used for signal evaluation per distance data point, the corresponding evaluation bandwidth $B_{{{\text{eval}}}}$, and the percentage of accepted data points. The upper plot shows the Allan deviation as a function of distance averaging time $\tau$ for all five configurations, and the lower diagram depicts the standard deviation. Deviations of less than 2 µm were demonstrated at record-high effective measurement rates of up to 495 MHz. (**b**) Same as (**a**), but with an optical booster amplifier (EDFA), which increases the tolerance with respect to optical losses and therefore permits a longer measurement reach compared to (**a**). At an effective measurement rate of 10 kHz, we demonstrate reliable ranging with standard deviations of less than 1 µm for free-space losses of more than 70 dB.

To quantify the sensitivity of our system with respect to low optical return power, we characterize the Allan deviation and the standard deviation of the measured distances for three different optical power levels, which are adjusted by introducing attenuators and neutral-density (ND) filters in the free-space beam path. The table in Fig. 3a lists the parameters of the three measurements, i.e., the optical return power, the free-space loss, the number $N_{{{\text{eval}}}}$ of pulse repetition periods per distance data point, and the corresponding evaluation bandwidth $B_{{{\text{eval}}}} = {1 \mathord{\left/ {\vphantom {1 {\left( {N_{{{\text{eval}}}} T_{{\text{r}}} } \right)}}} \right. \kern-\nulldelimiterspace} {\left( {N_{{{\text{eval}}}} T_{{\text{r}}} } \right)}}$ along with the percentage of accepted data points. Note that the evaluation bandwidth may slightly vary between the measurements due to a slow drift of the FSR difference $\delta \omega_{{\text{r}}} = {{2\pi } \mathord{\left/ {\vphantom {{2\pi } {T_{{\text{r}}} }}} \right. \kern-\nulldelimiterspace} {T_{{\text{r}}} }}$.

The lowest return power in our experiments is – 40 dBm. At this power level, the beat signals in the electric spectrum are barely detectable, and we did not included the recordings at maximum bandwidth ($N_{{{\text{eval}}}} = 1$), which are subject to large standard deviations of 500 µm or more with less than 5% of the data points accepted according to the criteria discussed after Eq. (5). The upper graph of Fig. 3a shows the Allan deviation as a function of averaging time $\tau = N_{{{\text{av}}}} N_{{{\text{eval}}}} T_{{\text{r}}}$. For the highest optical return power and for a measurement bandwidth of $B_{{{\text{meas}}}} = B_{{{\text{eval}}}} = 495\;{\text{MHz}}$ (blue curve), the Allan deviation for $\tau = 2.02\;{\text{ns}}$ ($N_{{{\text{av}}}} = 1$) amounts to 1.67 µm (blue circle). To the best of our knowledge, this is the highest measurement bandwidth demonstrated with any ranging system. Specifically, this measurement bandwidth can well compete with recently demonstrated values of up to 400 MHz, which were achieved with highly stable, but bulky and technically complex mode-locked fiber lasers^30,31^. When averaging over $N_{{{\text{av}}}} > 1$ consecutively measured distances, the Allan deviation decreases to $\sigma_{{\text{A}}} = 23\;{\text{nm}}$ for an effective measurement rate of $B_{{{\text{meas}}}} = 9.87\;{\text{kHz}}$ ($\tau = 101\;\upmu {\text{s}},\,\,N_{{{\text{av}}}} = 50119,\,\,B_{{{\text{eval}}}} = 495\;{\text{MHz}}$). Similar results are achieved when the same data record is evaluated at $B_{{\text{eval}}} = 49.5\;{\text{MHz}}$ (red curve) beginning at $\tau = 20.2\;{\text{ns}}$ with $N_{{{\text{av}}}} = 1$. The red and the blue curves approximately coincide, confirming that averaging over, e.g., $N_{{{\text{av}}}} = 10$ distance samples subsequently acquired at a high evaluation bandwidth and sufficiently high return power leads to approximately the same result as tenfold increased evaluation period $T_{{{\text{eval}}}}$ for each distance measurement.

Reducing the optical return power leads to an increase of the Allan deviation for all averaging times and evaluation bandwidths. At – 20 dBm optical return power (green and magenta line), the Allan deviation increases to 3.9 µm for an effective measurement rate of $B_{{{\text{meas}}}} = B_{{{\text{eval}}}} = 497\;{\text{MHz}}$ ($\tau = 2.01\;{\text{ns}}$, $N_{{{\text{av}}}} = 1$), and reduces to 26 nm for $B_{{{\text{meas}}}} = 9.91\;{\text{kHz}}$ ($\tau = 101\;\upmu {\text{s}}$, $N_{{{\text{av}}}} = 50{\mkern 1mu} {\mkern 1mu} 119$). At even lower return power levels, proper phase unwrapping for distance reconstruction according to Eq. (4) is not possible at the highest evaluation bandwidths, since the accuracy of the phase differences $\delta \Phi_{\mu } (d)$ suffers from electrical noise. To still obtain reliable distance values at a return power of – 40 dBm, we reduce the evaluation bandwidth to $B_{{\text{eval}}} = {1 \mathord{\left/ {\vphantom {1 {\left( {10{\mkern 1mu} T_{{\text{r}}} } \right)}}} \right. \kern-\nulldelimiterspace} {\left( {10{\mkern 1mu} T_{{\text{r}}} } \right)}} = 48.7\;{\text{MHz}}$ (orange curve). To the best or our knowledge, this represents the highest loss tolerance demonstrated so far for dual-comb distance metrology that fully relies on chip-scale frequency comb generators. We believe that the loss tolerance of such systems can be further improved, considering the outstanding sensitivity levels that have been demonstrated for ranging with comb sources built from fiber-optic or discrete components^32–35^. At $B_{{{\text{meas}}}} = B_{{{\text{eval}}}}$ ($\tau = 20.5\;{\text{ns}} ,$ $N_{{{\text{av}}}} = 1$), i.e., without averaging of subsequently acquired distance samples, the Allan deviation amounts to 16 µm, and reduces to 0.26 μm for $\tau = 101\;\upmu {\text{s}}$ ($N_{{{\text{av}}}} = 4{\mkern 1mu} {\mkern 1mu} 898$), corresponding to an effective measurement rate of $B_{{{\text{meas}}}} = 9.95\;{\text{kHz}}$.

The lower graph of Fig. 3a shows the standard deviation of the distance measurements with the same color coding as in the upper graph. Allan deviation and standard deviation are nearly identical. This indicates that the distance measurement errors can be described by spectrally white noise and are not impaired by any drift processes^29^. For an optical return power of 7 dBm, orange line, the measurement accuracy is limited by the noise floor of our ADC, whereas shot noise and the thermal noise of the detector electronics represent the dominant limitation for the lower received power levels of $- 20\;{\text{dBm}}$ and $- 40\;{\text{dBm}}$. The theoretically achievable precision of all measurements is approximately a factor of $3 \ldots 10$ better than the values we demonstrated here, indicating that the system can benefit from a further optimized implementation. A more detailed discussion can be found in the Supplementary Information Sections ‘Noise impairments of recorded signals’ and ‘Impact of shot noise on the measurement precision’.

In a second set of experiments, we boost the SI comb power in the receiver by an EDFA and repeat the measurements for larger target distances corresponding to higher free-space losses. To avoid damaging the reference balanced photodetector (BD_R_) depicted in Fig. 2a, we additionally include a variable optical attenuator (VOA) between the 90/10 coupler after the EDFA and the 50/50 coupler before BD_R_. We set the attenuation at the VOA such that the power of the SI comb reaching BD_R_ is approximately 0 dBm, which is sufficient for the detection of reference beat signals and read-out of the corresponding phase. The measurement and evaluation parameters are again listed in the table at the top of Fig. 3b, and the corresponding Allan deviations and standard deviations are shown in the graphs below the table. At highest optical return powers of + 5 dBm (26 dB free-space loss), the Allan deviation increases by approximately a factor of 2 compared to the measurement without an EDFA. We attribute this to the ASE noise of the EDFA, for which a noise figure of approximately 5 dB is specified by the manufacturer. At lower optical return powers, however, both the Allan deviation and the standard deviation become comparable to the measurement without an EDFA, see Fig. 3a,b (orange curves), which supports the notion that the phase measurement errors in this case are dominated by shot noise caused by the LO comb and by thermal noise of the receiver electronics, see Section ‘Noise impairments of recorded signals’ of the Supplementary Information for a more detailed analysis. An Allan deviation of 0.26 µm for an effective measurement rate of $B_{{{\text{meas}}}} = 9.95\;{\text{kHz}}$ ($\tau = 100\;\upmu {\text{s}}$, $N_{{{\text{av}}}} = 5{\mkern 1mu} {\mkern 1mu} 012$) is achieved at a free-space loss of 71 dB. Note that the standard deviation of the measurement at + 5 dBm (Fig. 3b, lower graph, blue and red curve) does not continuously decrease when increasing the averaging time, but reaches a plateau of $\sigma = 70\;nm$ near $\tau = 100\;\upmu {\text{s}}$. We relate this to a drift of the optical path lengths in our setup during this specific measurement.

Next we move the target mirror in Fig. 2a with a feedback-stabilized stage (Physik Instrumente, M511.HD) to $N_{{\text{p}}} = 16$ positions and record the distances obtained with our ranging system. The $N_{{\text{p}}} = 16$ mirror positions are evenly spaced by $\Delta z = 200\;\upmu {\text{m}}$, and the absolute positioning accuracy of the stage is specified to be better than 50 nm. The range of distances covers the full unambiguity distance of our system. To eliminate the impact of fiber drift on the measured distance^7^, we periodically compare the measured free-space distance $d_{{{\text{tar}}}}$ to the target mirror with the distance $d_{{{\text{fix}}}}$ to a second fixed reference mirror by alternating the measurement paths at a rate of 2 kHz, see the Supplementary Information, Section ‘Free-space optical setup for compensation of fiber drift’ for details of the underlying setup. At each mirror position, $4970$ distance values are acquired for $d_{{{\text{tar}}}}$ and $d_{{{\text{fix}}}}$ over a period of 100 µs at an evaluation bandwidth of $B_{{{\text{eval}}}} = {1 \mathord{\left/ {\vphantom {1 {\left( {10{\mkern 1mu} {\text{T}}_{{\text{r}}} } \right) \approx 49.7\;{\text{MHz}} }}} \right. \kern-\nulldelimiterspace} {\left( {10{\mkern 1mu} {\text{T}}_{{\text{r}}} } \right) \approx 49.7\;{\text{MHz}} }}$. Out of these 4970 measurements, a number of $N_{{{\text{accept}}}} \approx 4100$ values are accepted based on the associated fit errors $\varepsilon \left( {d_{i} } \right)$, Eq. (5). An example of measured and evaluated data can be found in the Supplementary Information, Fig. S5. For evaluating the accuracy of the ranging system, we first calculate the measured position $z_{{{\text{tar}}}}$ of the target mirror at each of the $N_{{\text{p}}} = 16$ stage positions, which is given by the path-length differences of the individual measurements. In the following, the mirror position is indicated by a subscript $m = 1 \ldots N_{{\text{p}}} ,$ and a subscript $l = 1 \ldots N_{{{\text{accept}}}}$ is used to refer to the individual pairs of measured distances to the target and the reference mirror, $z_{{{\text{tar,}}m,l}} = d_{{{\text{tar}},m,l}} - d_{{{\text{fix,}}l}}$. To quantify the precision and the accuracy of our ranging system, we first calculate the average of the measured target position $\overline{z}_{{{\text{tar,}}m}}$ for each stage position *m* along with the associated measurement uncertainty, quantified by the standard deviation $\sigma_{{z_{{{\text{tar}}}} ,m}}$,

10 $$\begin{aligned} \overline{z}_{{{\text{tar,}}m}} & = \frac{1}{{N_{{{\text{accept}}}} }}\mathop \sum \limits_{l = 1}^{{N_{{{\text{accept}}}} }} d_{{{\text{tar}},m,l}} - d_{{{\text{fix,}}l}} , \\ \sigma_{{z_{{{\text{tar}}}} ,m}}^{2} & = \frac{1}{{N_{{{\text{accept}}}} - 1}}\sum\limits_{l = 1}^{{N_{{{\text{accept}}}} {\kern 1pt} }} {\left( {z_{{{\text{tar}},m,l}} - \overline{z}_{{{\text{tar,}}m}} } \right)^{2} } . \\ \end{aligned}$$

Each of the measured target-mirror positions is associated with a nominal position $z_{{{\text{stage,}}m}} = m{\mkern 1mu} \Delta z + z_{0}$ of the mirror as set by the translation stage, where $z_{0}$ accounts for a constant offset $\overline{z}_{{{\text{tar,}}m}}$ between the *z*-scale of our ranging system and the *z*-scale of the stage encoder. For each mirror position *m*, we then calculate the distance error, i.e., the deviation of averaged measured mirror positions $\overline{z}_{{{\text{tar,}}m}}$ to the nominal positions $z_{{{\text{stage,}}m}}$ set by the stage,

11 $$\varepsilon_{{{{z,}}m}} = \overline{z}_{{{\text{tar,}}m}} - m\,\Delta z - z_{0} .$$

For simplicity, the constant offset $z_{0}$ is chosen to achieve a zero-mean deviation of the nominal mirror position from its measured counterpart when averaging over all of the $N_{{\text{p}}}$ mirror positions, $\sum\nolimits_{m = 1}^{{N_{{\text{p}}} }} {\varepsilon_{{z{,}m}} } = 0$.

**Figure 4:**
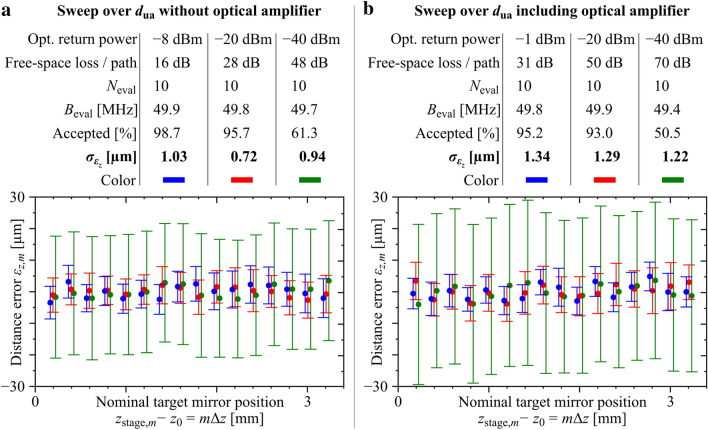
Unambiguity-distance sweep for varying free-space losses with and without EDFA, see Fig. 2a for the underlying experimental setup and Section ‘Detailed description of experimental setups’ of the Supplementary Information for further details. (**a**) Unambiguity-distance sweep without optical amplifier. The table lists the optical return power, the associated optical loss per path, the amount of considered pulse repetition periods per distance data point $N_{{{\text{eval}}}}$, the corresponding evaluation bandwidth $B_{{{\text{eval}}}}$ averaged over the measurement series, the percentage of accepted data points, and the variance $\sigma_{{\varepsilon_{{\text{z}}} }}$ of the distance errors $\varepsilon_{{z{,}m}}$ for three different measurement series. The figure shows the residual distance error of each target mirror position, i.e., the difference between the set and the mean of the measured position as a function of the nominal target mirror position $z_{{{\text{stage,}}m}} - z_{0} = m\Delta z$ as set by the stage over the unambiguity distance of the system. Error bars indicate the standard deviation $\sigma_{{z_{{{\text{tar}}}} ,m}}$ of the point-wise computed difference between the beam-path length to the static reference mirror and the beam-path length to the target mirror. For better readability, the data points and error bars belonging to the same position $z_{{{\text{stage,}}m}} - z_{0} = m\Delta z,{\mkern 1mu} {\mkern 1mu} m = 1 \ldots 16,{\mkern 1mu} {\mkern 1mu} \Delta z = 200\;\mu {\text{m}}$ of the target mirror are slightly offset horizontally with respect to each other. (**b**) Same as (**a**), but with an EDFA acting as optical booster amplifier. Further details on the experiment and the data evaluation can be found in Section ‘Evaluation of unambiguity-distance sweep with fiber drift compensation’ of the Supplementary Information.

To quantify the performance of our ranging system, we extract the distance error $\varepsilon_{{z{,}m}}$ along with the associated measurement uncertainty $\sigma_{{z_{{{\text{tar}}}} ,m}}$ at each mirror position *m*. This procedure is repeated for a wide range of optical return powers with and without EDFA, see Fig. 4. For each measurement, the return power and the evaluation parameters are listed in the tables on the top of Fig. 4a,b. The plots below these tables show the distance errors $\varepsilon_{{{z,m}}}$ according to Eq. (11) as a function of the target position $m{\mkern 1mu} \Delta z$, recorded over a full unambiguity distance $d_{{{\text{ua}}}} = 3.01\;{\text{mm}}$ centered at $z_{0} \approx 1{\text{m}}$. The error bars represent the standard deviations $\sigma_{{z_{{{\text{tar}}}} ,m}}$ according to Eq. (10). We do not observe any outliers throughout our measurements, which demonstrates the reliability of the approach. Moreover, we do no observe any cyclic errors, which would lead to a systematic variation of $\varepsilon_{z,m}$ over the unambiguity distance. For the system without optical amplifier, we find measurement uncertainties $\sigma_{{z_{{{\text{tar}}}} ,m}}$ of approximately 19 µm even for return power levels as low as – 40 dBm. For the system with optical amplifier, the measurement uncertainties $\sigma_{{z_{{{\text{tar}}}} ,m}}$ increase to approximately 25 µm for the same power level, corresponding to a free-space loss of 70 dB.

As an additional performance metric, we calculate the variance $\sigma_{{\varepsilon_{z} }}^{2}$ of the distance errors $\varepsilon_{{z{,}m}}$ for the various mirror positions

12 $$\sigma_{{\varepsilon_{z} }}^{2} = \frac{1}{{N_{{\text{p}}} - 1}}\sum\limits_{m = 1}^{{N_{{\text{p}}} }} {\varepsilon_{z,m}^{2} } .$$

This figure is an indicator of the overall accuracy of ranging system and is specified in the second to last row of the tables in Fig. 4a,b. We find this number to be approximately 1 µm, merely independent of the optical return power. We attribute this observation to additional ranging errors which are caused by the periodic alternation between target and reference mirror, see Section ‘Evaluation of unambiguity-distance sweep with fiber drift compensation’ of the Supplementary Information, and which are independent of the return power levels. The quantity $\sigma_{{\varepsilon_{z} }}$ does hence not represent the fundamental accuracy limitation of our optical ranging system but is rather to be understood as an upper boundary of the achievable measurement accuracy, dictated by the specific experimental setup.

To overcome the limited unambiguity distance $d_{{{\text{ua}}}} = 3.01\;{\text{mm}}$ of our system, several approaches can be used. Evidently, it is always possible to combine the dual-comb scheme with a simple time-of-flight system for coarse ranging. Alternatively switching the role of the LO comb and the SI comb allows to greatly extend the unambiguity distance via the Vernier effect^9^. In another approach, the LO comb can also be sent to the target and the sum of the resulting phases detected at the balanced photodetectors can be evaluated^36^. These approaches allow for high-precision ranging over distances that are limited only by the coherence length of the QD-MLLD. For the devices used in our current experiments, the coherence length is of the order of tens of meters and can be increased further by applying linewidth-reduction techniques^14,17,27,28^.

### High-speed ranging

**Figure 5:**
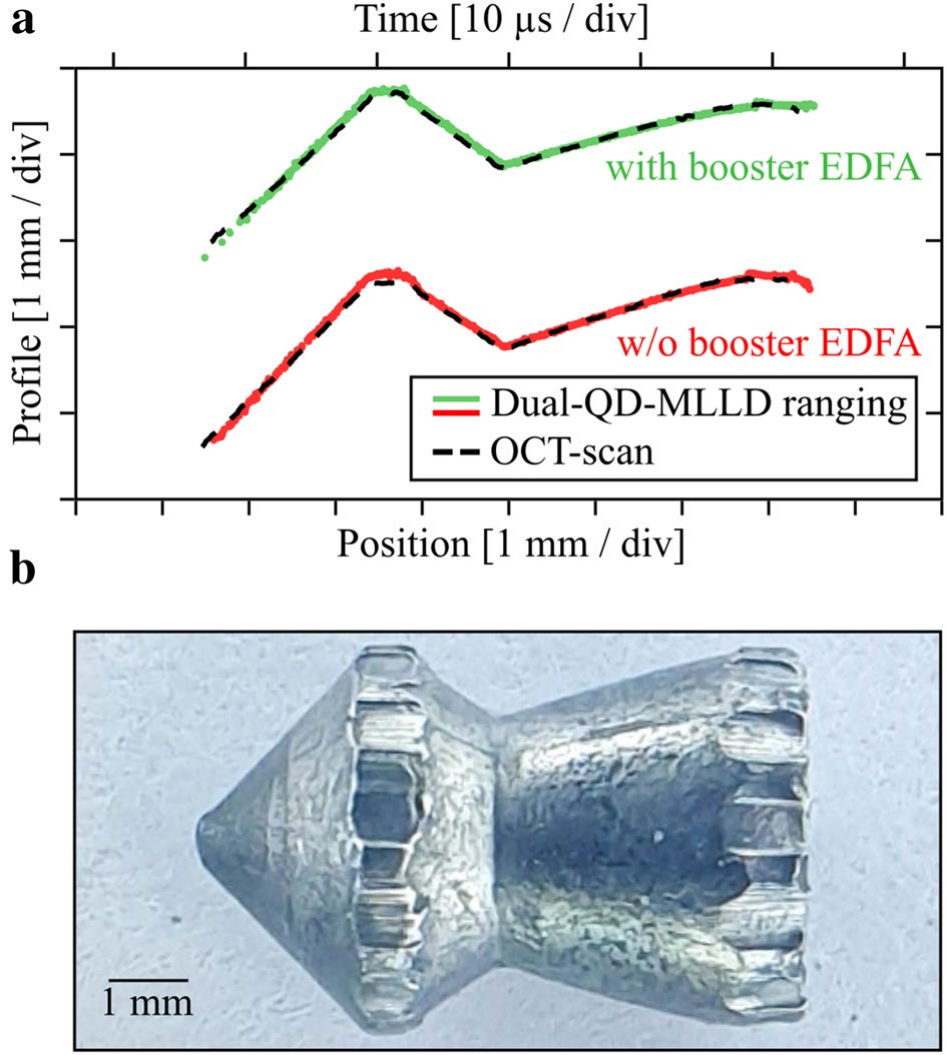
Surface profile measurements of air-gun projectile passing the measurement beam at a speed of approximately 150 m/s. (**a**) Surface profiles measured on the flying projectile, using the one-port ranging system, (Fig. S6 in the Supplementary Information), with EDFA (green) and without EDFA (red). The free-space signal beam power amounts to 9 dBm and 22 dBm for the measurement with and without EDFA, respectively. For better visibility, the red and the green curve are separated by a vertical offset. The black dashed curves denote the profiles of both investigated projectiles, obtained from an optical coherence tomography (OCT) measurement on the static projectile after recovery from the back-stop. (**b**) Photograph of the projectile measured in (**a**), red curve.

To demonstrate the ultrafast-sampling capabilities of our ranging system, we measure the profile of a flying air-gun projectile. To simplify free-space beam alignment, we replace the two separated collimators of Fig. 2a with a single collimator and a fiber-optic circulator, see Section ‘One-port ranging system and triggering data acquisition of projectile measurements’ of the Supplementary Information for further details of the experiment. We focus the free-space beam at the anticipated projectile trajectory. In Fig. 5a, we depict the recorded profile of a projectile that is shot through the measurement beam at a speed of approximately $150\;{\text{m}} \,{\text{s}}^{ - 1}$. We use a measurement bandwidth of $B_{{{\text{meas}}}} = B_{{{\text{eval}}}} = {1 \mathord{\left/ {\vphantom {1 {\left( {10T_{{\text{r}}} } \right)}}} \right. \kern-\nulldelimiterspace} {\left( {10T_{{\text{r}}} } \right)}} = 49.1\;{\text{MHz}}$ and perform the experiment without an EDFA (red trace) and with EDFA (green trace). For the given projectile speed, this corresponds to a separation of neighboring sample points of approximately 3 µm. For the measurement without EDFA, an average of 61% of the evaluated distance data points are accepted using the fit-error criterion according to Eq. (5). The black dashed line on top of the red trace is the result of an optical coherence tomography (OCT) measurement that was performed on the static projectile after recovery from the back-stop. For better comparison, the OCT-based profile and the profile obtained from the flying projectile were rotated with respect to each other, and an actual speed of the projectile of 151 m s^−1^ was estimated for best agreement. The same procedure was performed for the measurement with EDFA, for which the signal comb power emitted from the collimator amounts to 22 dBm. In this experiment, 65% of the measured distance points are accepted, and a speed of 153 m s^−1^ was estimated by comparing the profile on the flying projectile to the OCT measurement. Figure 5b shows a photograph of the projectile used in the ranging experiments without EDFA.

## Discussion

We have demonstrated high-precision dual-comb ranging with quantum-dash mode-locked laser diodes (QD-MLLD) as particularly compact and efficient frequency comb sources. The devices offer easy operation by a simple DC drive current and provide spectrally flat frequency combs with line spacings of tens of gigahertz. We demonstrate measurement rates up to 500 MHz, corresponding to the highest measurement rate demonstrated with any ranging system so far. In comparison to other chip-scale comb sources, QD-MLLDs provide comparatively high comb line powers of the order of 500 µW—more than one order of magnitude higher than those of Kerr soliton frequency comb generators^37–39^. This leads to high tolerance with respect to optical loss in the free-space path of a ranging system. In our experiments, we find a high loss tolerance of 49 dB without optical amplifiers and of 71 dB in case a booster EDFA is used. To the best of our knowledge, this is the highest loss tolerance demonstrated so far for a comb-based measurement system that relies on chip-scale light sources. The loss tolerance can be further improved by increasing the free-space beam power and by reducing the measurement rate, see Sections ‘Noise impairments of recorded signals’ and ‘Impact of shot noise on the measurement precision’ of the Supplementary Information for a more detailed analysis of the noise limitations in dual-comb ranging systems. We demonstrate the measurement speed of our system by high-precision in-flight sampling of air-gun pellets moving at a speed of 150 m s^−1^. Based on our findings, we believe that quantum-dash mode-locked laser diodes (MLLD) are an attractive option for comb generation in compact power-efficient LiDAR systems.

## Supplementary Information

Supplementary Information.
