# Supplementary material for: Ultra-fast optical ranging using quantum-dash mode-locked laser diodes
Source: Sci Rep. 2022 Jan 20;12:1076. doi: 10.1038/s41598-021-04368-4 (PMC8776879; doi:10.1038/s41598-021-04368-4)
Supplement: Supplementary file 1 — Supplementary Information. [file 41598_2021_4368_MOESM1_ESM.pdf]

Ultra-fast optical ranging using quantum-dash mode-locked laser diodes

# - Supplementary Information -

Philipp Trocha,[1] Juned Nassir Kemal,[1] Quentin Gaimard,[2] Guy Aubin,[2] François Lelarge,[3] Abderrahim Ramdane,[2] Wolfgang Freude,[1] Sebastian Randel[1] and Christian Koos[1,4,*]

[1] Institute of Photonics and Quantum Electronics (IPQ), Karlsruhe Institute of Technology (KIT), Engesserstrasse 5, 76131 Karlsruhe, Germany
[2] Centre de Nanosciences et de Nanotechnologies CNRS, Université Paris-Saclay, Boulevard Thomas Gobert 10, 91220 Palaiseau, France
[3] Almae Technologies, Route de Nozay, 91460 Marcoussis, France
[4] Institute of Microstructure Technology (IMT), Karlsruhe Institute of Technology (KIT), Hermann-von-Helmholtz-Platz 1, 76344 Eggenstein-Leopoldshafen, Germany
[*] *Correspondence and requests for materials should be addressed to C. K. (phone: +49 721 608-42491, email: christian.koos@kit.edu)*

Free-space optical setup for compensation of fiber drift

For the ranging experiments described in Section 'System precision and accuracy' of the main manuscript, we compensate thermally and mechanically induced drift of the fiber lengths by periodically measuring the length of a fixed reference path and by comparing it to the measured path length to the target mirror. The corresponding setup is shown in Fig. S1. Light from the transmitter is guided to an optical output collimator (COL_O) and emitted as a free-space beam. Neutral-density filters (ATT) are used to adjust the power of the free-space beam. The beam is split into two parts at a beam splitter (BS), and both parts are guided towards the optical input collimator (COL_I) and from there to the receiver (Rx). The grey path serves as a fixed reference, whereas the red beam is guided towards the target mirror, reflected, and sent to the input collimator. A chopper wheel (CPW) alternatingly blocks the red and the grey beam with a frequency of 2 kHz. Comparing the path length d_{tar} to the fixed reference length d_{fix} on a timescale of less than a millisecond allows to eliminate drift of the fiber lengths, which can amount to several micrometers on a time-scale of minutes[1]. Note that we introduced a factor of 2 into the definition of the beam path lengths to account for the back- and forth-propagation to the target mirror. As a consequence, the overall length of the red beam path is denoted as $2d_{\text{tar}}$, whereas the overall length of the grey path is $2d_{\text{fix}}$. A movement of the target mirror by a small increment Δz hence leads to an equivalent change of the difference $d_{\text{tar}} - d_{\text{ref}}$. Further details regarding the components used in our experiments can be found in Supplementary Section 'Detailed description of experimental setups' below.

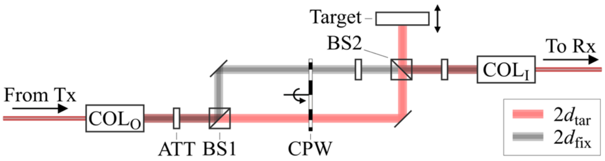

Figure S1: Free-space optical setup used for compensating thermally and mechanically induced drift of the fiber lengths during the ranging experiments. Light is emitted from the transmitter (Tx, left) and guided to an output collimator (COL_O) by optical fibers. The emitted light is guided to the receiver (Rx, right) via two different paths. One path serves as static reference with a fixed length $2d_{\text{fix}}$ (grey), while the beam following the other path (red) is guided towards the target, traveling a distance of $2d_{\text{tar}}$ before reaching the input collimator COL_I. A combination of beam splitters (BS) and a rotating chopper wheel (CPW) allows to alternate between the two paths with a frequency of 2 kHz. Comparing the distance d_{tar} to the fixed reference distance d_{fix} on a timescale of less than a millisecond allows to greatly reduce the impact of fiber-length drifts. Optical power levels can be adjusted via variable neutral density filters (ATT). Details on the components that were used in the setup can be found in Supplementary Section 'Detailed description of experimental setups' below.

Selection of reliable distance data by fit error

As described in Section 'System, operation principle and digital signal processing' of the main manuscript, every distance data point d is the result of a linear fit to a set of unwrapped phases $\delta\Phi_\mu$ of μ beat signals. The quality of the fit is given by the fit error ε defined in Eq. (5) of the main manuscript. For low received optical powers, the associated beat signals are weak and the extracted phases are distorted by shot noise of the LO comb, by thermal noise of the receiver electronics, and by noise of the analog-to-digital-converters, see Supplementary Sections 'Noise impairments of recorded signals' and 'Impact of shot noise on the measurement precision' below for details. As a result, the unwrapped phase values cannot be exactly fitted by a straight line and therefore the error ε of the linear fit will be high. To identify unreliable data points in a sequence of measured distances $d_i, i = 1,2,3\ldots$, we introduce an adaptive local threshold $\varepsilon_{\text{th,loc}}(d_i)$ that distinguishes between reliable ($\varepsilon(d_i) < \varepsilon_{\text{th,loc}}(d_i)$) and unreliable ($\varepsilon(d_i) \geq \varepsilon_{\text{th,loc}}(d_i)$) data points. The adaptive threshold $\varepsilon_{\text{th,loc}}(d_i)$ is defined by a moving average of the fit error over an interval of M measurements that is centered at the data point of interest d_i and by a constant offset γ,

$$\varepsilon_{\text{th,loc}}(d_i) = \overline{\varepsilon(d_i)} + \gamma, \qquad \overline{\varepsilon(d_i)} = \frac{1}{M} \sum_{m=i+\lfloor -M/2 \rfloor +1}^{i+\lfloor M/2 \rfloor} \varepsilon(d_m), \tag{S1}$$

where the floor operator $\lfloor \cdot \rfloor$ denotes the nearest smaller integer. The threshold offset accounts for the fact that the fit errors $\varepsilon(d_i)$ are approximately symmetrically distributed above and below the local moving average $\overline{\varepsilon(d_i)}$, and that data points with an error slightly above the local moving average can still be regarded reliable. Note that, when used in real-time systems, this procedure leads to a latency of $M/2$ data points. This can be avoided by averaging over M values of the fit errors $\varepsilon(d_i)$ that precede the actual data point of interest instead of averaging around this data point, as described in Eq. (S1). In addition to the adaptive local threshold $\varepsilon_{\text{th,loc}}(d_i)$, we introduce a global upper threshold $\varepsilon_{\text{th,up}}$ that is used in case the fit errors become too large. The upper

threshold accounts for the fact that, in case of very noisy data, the local moving average $\overline{\varepsilon(d_i)}$ may become too large and cannot be used as a base for determining the threshold for the acceptable fit error. Conversely, in case of high received power levels, the mean error can become very small, and there is no reason to reject any data points. This is taken into account by global lower threshold $\varepsilon_{\text{th,lo}}$. The overall threshold ε_{th} for the fit error of a data point d_i can hence be defined by

$$\varepsilon_{\text{th}}(d_i) = \begin{cases} \varepsilon_{\text{th,up}} & \text{for} \quad \varepsilon_{\text{th,loc}}(d_i) \geq \varepsilon_{\text{th,up}} \\ \varepsilon_{\text{th,lo}} & \text{for} \quad \varepsilon_{\text{th,loc}}(d_i) \leq \varepsilon_{\text{th,lo}} \\ \varepsilon_{\text{th,loc}}(d_i) & \text{else} \end{cases} . \tag{S2}$$

According to Eq. (5) of the main manuscript, all measurements with an error $\varepsilon(d_i) \geq \varepsilon_{\text{th}}(d_i)$ are discarded. In our evaluation, we chose $M = 101$, $\gamma = 0.01 \times 2\pi$, $\varepsilon_{\text{th,up}} = 0.16 \times 2\pi$ and $\varepsilon_{\text{th,lo}} = 0.02 \times 2\pi$, which were found to lead to a good trade-off between rejection of reliable and acceptance of unreliable data points over a large range of received power levels. The values for the global limits $\varepsilon_{\text{th,up}}$ and $\varepsilon_{\text{th,lo}}$ as well as the offset γ and the number M of samples in the moving average are fixed parameters of our measurement system, which may be adapted to the respective application, finding a favorable trade-off between the number of accepted data points, the ability of the system to quickly adapt to new overall levels of the fit error, and the quality of the obtained distance data points.

In Fig. S2 we show how this selection of distance data points affects the final result. Figure S2a shows the distance profile obtained from a flying projectile moving at a speed of $150\,\text{m}\,\text{s}^{-1}$ as a function of measurement time, see Section 'High-speed ranging' of the main manuscript for more details. The red dots indicate accepted distance values where $\varepsilon(d_i) < \varepsilon_{\text{th}}(d_i)$, and the blue dots represent discarded data points where $\varepsilon(d_i) \geq \varepsilon_{\text{th}}(d_i)$. The most unreliable distance data points d_i are found at the beginning of the measurement, i. e., at the tip of the projectile, where the scattering surface is strongly inclined with respect to the measurement beam axis and thus only a small fraction of the incident power re-enters the system. Figure S2b depicts the associated fit errors – the green curve indicates the fit-error thresholds $\varepsilon_{\text{th}}(d_i)$, whereas blue dots correspond to discarded and red dots to accepted distance data points.

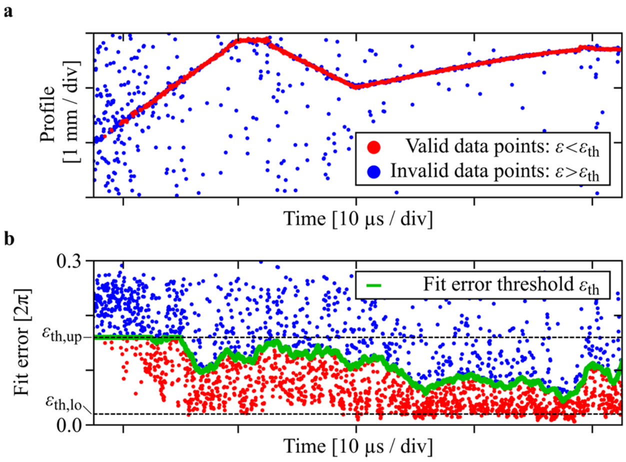

Figure S2: Distance data points and fit error for cross-sectional characterization of flying projectile, see Section 'High-speed ranging' of the main manuscript. **a** Distance data points as a function of measurement time. Red dots indicate accepted distance values and blue dots represent discarded data points. **b** Fit error associated with the various distance data points. Red points below the fit-error threshold $\varepsilon_{\text{th}}(d_i)$ according to Eq. (S2) (green curve) are considered accepted, whereas blue dots above the green line are discarded. The horizontal dashed lines indicate the global upper and lower thresholds $\varepsilon_{\text{th,up}} = 0.16 \times 2\pi$ and $\varepsilon_{\text{th,lo}} = 0.02 \times 2\pi$ for the fit error.

Noise impairments of recorded signals

In the following we investigate the impact of noise on our recorded signals and on the extracted distance values described in Sections 'System precision and accuracy' and 'High-speed ranging' of the main manuscript, and we identify the most relevant noise sources. To this end, we assume that the receiver of each optical signal consists of a balanced photodetector (BD), an electrical amplifier (EA) as well as an analogue-to-digital converter (ADC), see Fig. S3. We assume that the gain of the electrical amplifier is automatically controlled such that the full voltage range of the ADC is used during the recording of the signals. The photodetector is characterized by its responsivity $\mathcal{R}$, and the electrical amplifier by its gain *G* and its noise figure *F*.

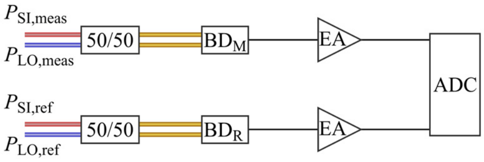

Figure S3: Model of the receiver system. The optical powers of the signal (SI) and the local oscillator (LO) comb on the balanced measurement and reference photodetectors $\mathrm{BD_M}$ and $\mathrm{BD_R}$ are denoted as $P_{\mathrm{SI,meas}}$, $P_{\mathrm{LO,meas}}$, $P_{\mathrm{SI,ref}}$ and $P_{\mathrm{LO,ref}}$, respectively. The photodetector output signals are fed to electrical amplifiers (EA) and recorded by a pair of analogue-to-digital converters (ADC). Noise may arise from the inherent shot noise of the photodetection process, from thermal noise of the electric amplifier, and from quantization noise of the ADC.

In our distance measurements, we determine the phase of beat notes in RF signals generated by the measurement and the reference photodetectors. For simplicity, we assume that the power of the signal and the LO optical comb P_{SI} and P_{LO} is evenly distributed across all N_{c} comb lines, such that the power per line amounts to $P_{\mathrm{SI}}/N_{\mathrm{c}}$ and $P_{\mathrm{LO}}/N_{\mathrm{c}}$, respectively. For a given photodetector responsivity $\mathcal{R}$ and a system impedance of $Z = 50\Omega$, the RF power P_{RF} of the beat signals on the reference (subscript 'ref') and the measurement (subscript 'meas') photodetector can be written as[1]

$$P_{\mathrm{RF,meas}} = \frac{Z}{2}\frac{2\mathcal{R}P_{\mathrm{SI,meas}}}{N_{\mathrm{c}}}\times\frac{2\mathcal{R}P_{\mathrm{LO,meas}}}{N_{\mathrm{c}}}, \tag{S3}$$

$$P_{\mathrm{RF,ref}} = \frac{Z}{2}\frac{2\mathcal{R}P_{\mathrm{SI,ref}}}{N_{\mathrm{c}}}\times\frac{2\mathcal{R}P_{\mathrm{LO,ref}}}{N_{\mathrm{c}}}. \tag{S4}$$

In the following, we estimate the ranging accuracy that can be achieved for low levels of received optical power on the measurement detector. In this case, the associated beat signal on the measurement detector is weak and heavily impaired by noise. We may hence restrict our analysis to the measurement detector, while assuming a 'perfect' beat signal on the reference detector. We further assume that the recorded RF signals are only impaired by shot noise with RF power $P_{\mathrm{n,shot}}$ and by thermal noise with power $P_{\mathrm{n,ther}}$. The associated signal-to-noise ratio (SNR) of the photocurrent from the measurement detector then reads

$$\mathrm{SNR} = \mathrm{SNR}_{\mathrm{RF,meas}} = \frac{P_{\mathrm{RF,meas}}}{P_{\mathrm{n,shot}} + P_{\mathrm{n,ther}}}. \tag{S5}$$

The shot noise is dominated by the contribution of the LO comb, which usually contributes a much higher optical power on the photodetector than the SI comb, $P_{\mathrm{SI}} \ll P_{\mathrm{LO}}$. Given a matched termination with an impedance $Z = 50\Omega$ and a noise bandwidth Δf which is approximately equal to the evaluation bandwidth B_{eval}, $\Delta f \approx B_{\mathrm{eval}}$, the shot-noise power after amplification can be written as

$$P_{\mathrm{n,shot}} \approx Z \times 2e\mathcal{R}P_{\mathrm{LO}}\Delta f \times G, \tag{S6}$$

where *e* is the elementary charge.

Thermal noise is generated in the internal 50 Ω impedance of the photodetector circuit and in the subsequent electrical amplifier. For a given system temperature T and an amplifier noise figure F, the total thermal noise power at the amplifier output can be written as

$$P_{\mathrm{n,ther}} = k_{\mathrm{B}} T\, \Delta f \times G \times F\,, \tag{S7}$$

where k_{B} denotes the Boltzmann constant.

Comparing Eqs. (S6) and (S7) shows that the system can always be operated in its shot-noise limited regime, $P_{\mathrm{n,shot}} > P_{\mathrm{n,ther}}$, provided that the LO power is sufficiently high. Specifically, for a photodiode responsivity of $\mathcal{R} = 0.5\,\mathrm{A\,W^{-1}}$, an amplifier noise figure of $F = 3$ (5 dB), and a system operated at room temperature ($k_{\mathrm{B}}T = 25\,\mathrm{meV}$), we find that shot-noise limited reception is already achieved for comparatively low LO comb powers of $P_{\mathrm{LO}} > 2\,\mathrm{mW}$. This holds independently of the amplifier gain G and the noise bandwidth Δf . For shot-noise limited reception, the SNR of the RF signal at the output of the electrical amplifier amounts to

$$\mathrm{SNR} \approx \frac{P_{\mathrm{RF}}}{P_{\mathrm{n,shot}}} = \frac{\mathcal{R}\, P_{\mathrm{SI}}}{N_{\mathrm{c}}^{2} e \Delta f.} \tag{S8}$$

For low received optical signal powers P_{SI}, the SNR according to Eq. (S8) can be directly translated into the precision of the distance measurement, see Supplementary Section 'Impact of shot noise on the measurement precision' below, provided that quantization noise and other impairments of the ADC can be neglected. In most cases of practical interest, this is a valid assumption: State-of-the-art ADC with bandwidths of a few GHz offer effective numbers of bits (ENOB) of approximately 10, which translates into an SNR of $(6.02\,\mathrm{ENOB} + 1.76)\,\mathrm{dB} = 62\,\mathrm{dB}$ that can ultimately be achieved for the digitized signal, provided that the received signal power is high enough and that the only relevant signal impairments are those coming from the ADC. However, this case is not of too much practical interest. Assuming an ENOB of 10, a responsivity of $\mathcal{R} = 0.5\,\mathrm{A\,W^{-1}}$, a comb line number of $N_{\mathrm{c}} = 25$ and a measurement rate of $\Delta f = 5\,\mathrm{MHz}$, ADC impairments would only become visible for relatively high received signal comb powers of more than 1 mW. For typically received power levels in the microwatt or nanowatt regime, the ranging performance of a properly designed system will thus be limited by shot noise.

We also analyze the different noise contributions found in our measurements. To this end, we extract the power spectral density of the spectrally white background noise that is found in the recorded RF beat signals for different received signal comb powers $P_{\mathrm{SI,meas}}$, see orange dots in Fig. S4a. To quantify the background noise level, we calculate the mean power spectral density in a frequency range of 24 to 26 GHz, where no beat signals are found, see orange box in Fig. S4b. For comparison, we also extract the expected ADC noise level (red dots) as well as the expected shot noise level (light grey dots). The ADC noise level is extracted from the ENOB of 5.0 that is specified by the manufacturer of the high-speed oscilloscopes (Keysight UXR0804A). The ADC acquisition range is adapted to the amplitude of the RF beat signals, and the ADC noise level thus decreases for lower signal comb powers until the smallest acquisition range is reached at a signal power of approximately $-35\,\mathrm{dBm}$. The shot noise is dominated by the contribution of the LO comb $P_{\mathrm{LO,meas}}$ of $7\,\mathrm{dBm}$ and does hence not vary significantly with the signal comb power. Note that in this set of measurements, no optical amplifier is used. The estimated shot-noise levels consider an effective gain figure G_{eff} of the RF amplifier, which also contains signal losses occurring in our electronic receiver components and which was obtained by comparing the expected beat signal power levels on the measurement detector, see Eq. (S3), with the recorded signals in our oscilloscopes. In addition, we consider an additional increase of the measured shot-noise level by approximately $3\,\mathrm{dB}$ due to fact that the high-speed oscilloscopes were operated at a sampling rate of only 128 GSa/s with an analogue anti-aliasing filter having a bandwidth of approximately 100 GHz. We find that for high received signal powers of $-10\,\mathrm{dBm}$ or more, the observed noise in our experiments can be fully explained with the expected ADC noise, see Fig. S4a. For signal comb powers below $-10\,\mathrm{dBm}$, the measured noise reaches a plateau at a power spectral density of approximately $-166.5\,\mathrm{dB\,V^{2}\,Hz^{-1}}$. We attribute this noise floor to a combination of shot noise and thermal noise of our RF amplifier. To confirm this notion, we also extract the power spectral density of approximately $-173\,\mathrm{dB\,V^{2}\,Hz^{-1}}$ that is to be expected for the thermal noise of the RF amplifier (SHF 807), see grey line in Fig. S4a, and we add it to the shot-noise level (grey dots) to obtain the overall noise floor of approximately $-167.5\,\mathrm{dB\,V^{2}\,Hz^{-1}}$ (dark grey dots). The thermal noise level was estimated from the noise figure of the

amplifier ($F = 6\,\mathrm{dB}$)[2], using again the effective gain G_{eff} of our electronic signal chain and taking again into account an additional increase of the measured noise by approximately $3\,\mathrm{dB}$ due to fact that the anti-aliasing filter was too broadband for the given sampling rate. We find that the noise level extracted from our measurements approaches the overall expected noise limit comprising both thermal and shot noise (dark grey dots) for low received power signal power levels, and we thus conclude that a combination of shot noise and thermal noise of the RF amplifier represents the main limitation of our system for low received signal power levels. Based on our analysis, we believe that the performance presented in our main manuscript can be further improved by using ADC with higher ENOB and RF amplifiers with lower noise figure, rendering thermal noise fully negligible compared to shot noise. In the following section, we estimate the achievable measurement precision for this scenario.

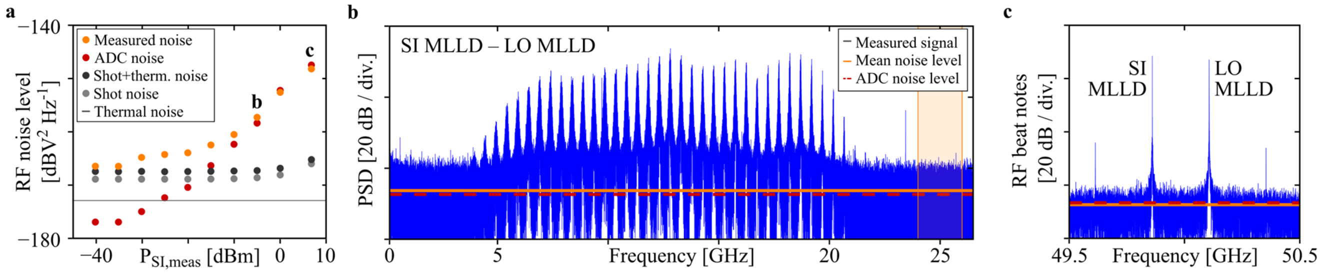

Figure S4: Analysis of the different noise contributions found in our measurements. **a** Power spectral density levels of the measured noise (orange dots), ADC noise (red dots), thermal noise (grey line), shot noise (light grey dots) and the sum of thermal noise and shot noise (dark grey dots) for different received signal powers incident on the measurement detector. The labels b and c refer to the measurements depicted in the respective subfigure. **b** Power spectral density (PSD) of an electric signal obtained from the measurement photodetector $\mathrm{BD_M}$ as shown in Fig. 2b of the main manuscript. The orange line indicates the mean noise level extracted in the frequency range 24 – 26 GHz (orange box), and the red dashed lines represents the expected ADC noise floor. For the incident signal comb power of −5 dBm, the noise floor is clearly limited by ADC noise. **c** RF beat notes of both MLLDs (blue), shown in Fig. 1e of the main manuscript. The mean noise floor (orange line) and the expected ADC noise floor (red dashed line) are again indicated by an orange and a red dashed line, respectively. The measured spectrum was corrected by a constant offset which compensates the decay of the frequency response of the oscilloscope towards higher frequencies.

Impact of shot noise on the measurement precision

In Ref. 1, we derived in detail how shot noise is related to the precision of a dual-comb ranging system, and we only summarize the main findings here to further analyze the performance limitations of our measurement system, see Section ‘System precision and accuracy’ of the main manuscript. To extract a distance value d_i from the measured signals, a straight line is fitted to a set of $\mu = 1, 2, \ldots, N_{\mathrm{b}}$ unwrapped phase differences $\delta\Phi_\mu(d_i) = \Phi_{\mu,\mathrm{meas}}(d_i) - \Phi_{\mu,\mathrm{ref},i}$. These phase differences are impaired by random fluctuations, which we assume to be uncorrelated and to have the same distribution with zero mean and with a standard deviation $\sigma_{\delta\Phi}$. The standard deviation σ of the extracted distance, as obtained from a linear regression of N_{b} phase difference values, is then given by

$$\sigma = \sqrt{\frac{12}{N_{\mathrm{b}}^3}} \frac{c_0}{2\omega_{\mathrm{SI,r}}\, n_{\mathrm{air}}} \times \sigma_{\delta\Phi}, \tag{S9}$$

see Eq. (S26) in Ref. 1. Note that it may be advantageous to use only a subset of N_{b} comb lines of the overall N_{c} available lines for extracting the distance information and to discard some lines that are strongly impaired by noise. The standard deviation $\sigma_{\delta\Phi}$ of the phase differences is estimated from the standard deviation $\sigma_{\Phi,\mathrm{meas}}$ of the phases obtained from the signal of the measurement photodetector and from the standard deviation $\sigma_{\Phi,\mathrm{ref}}$ of the phases obtained from the reference photodetector. The quantities $\sigma_{\Phi,\mathrm{meas}}$ and $\sigma_{\Phi,\mathrm{ref}}$ depend on the respective signal-to-noise ratio (SNR)[3],

$$\sigma_{\delta\Phi}^2 = \sigma_{\Phi,\mathrm{meas}}^2 + \sigma_{\Phi,\mathrm{ref}}^2 = \frac{1}{2\times \mathrm{SNR}_{\mathrm{meas}}} + \frac{1}{2\times \mathrm{SNR}_{\mathrm{ref}}}. \tag{S10}$$

Note that the relation between the phase variance and the SNR on the respective detector is only valid for high $\mathrm{SNR} > 10$ and needs to be replaced with a more precise expression for lower SNR, see Eq. (17) in Ref. 2. Note also that Eq. (S10) accounts for the finite SNR of both the reference and the measurement detector. For low received optical power, the standard deviation $\sigma_{\delta\Phi}$

of the phase differences will be dominated by the shot noise on the measurement detector, see Supplementary Section 'Noise impairments of recorded signals' for details.

To estimate $\mathrm{SNR}_{\mathrm{meas}}$, we rely on Eqs. (S5) and (S6), and equivalent relations can be used for estimating $\mathrm{SNR}_{\mathrm{ref}}$. In both cases, we assume that the LO power is high enough such that thermal noise of the respective receiver circuit, Eq. (S7) can be neglected. If, in addition, an optical amplifier is used to boost the SI comb power, additional amplified spontaneous emission (ASE) noise will further degrade the SNR performance. This can be taken into account by replacing the relation for the shot-noise power at the measurement detector, Eq. (S5), by an equivalent relation that includes for the noise figure F_{EDFA} of the EDFA,

$$\mathrm{SNR}_{\mathrm{meas}} \approx \frac{P_{\mathrm{RF}}}{P_{\mathrm{n,shot}} \times F_{\mathrm{EDFA}}}. \tag{S11}$$

For the EDFA used in our experiments, a noise figure of 5.4 dB ($F_{\mathrm{EDFA}} = 3.5$) is specified by the manufacturer.

Inserting the SNR for the measurement and the reference detector in Eq. (S10), we can use Eq. (S9) to determine the achievable precision for given comb parameters $P_{\mathrm{SI,meas}}$, $P_{\mathrm{LO,meas}}$, $P_{\mathrm{SI,ref}}$, $P_{\mathrm{LO,ref}}$, $\omega_{\mathrm{SI,r}}$, N_{b}, N_{c}, a given responsivity $\mathcal{R}$ of the photodetectors, and a given electrical bandwidth Δf. The results are summarized in Table S1, specifying the achievable shot-noise limited precision σ for the different setup configurations that are described in Fig. 3 of the main manuscript. Note that, for the sake of simplicity, we have rounded some of the parameters listed in Table S1 such as the FSR $\omega_{\mathrm{SI,r}}$ – the precise values can be found in the main manuscript. For data evaluation, we choose $N_{\mathrm{b}} = 26$ comb lines which proved to work best at low optical return powers of the signal comb, whereas the entire comb consisted of approximately $N_{\mathrm{c}} = 40$ lines, see also Fig. 1 in the main manuscript. The electrical bandwidth Δf was assumed to be equal to the evaluation bandwidth B_{eval}.

Table S1: Achievable measurement precision limited by shot noise. Experimental parameters are rounded for simplicity.

**Measurement No.**	**1**	**2**	**3**	**4**	**5**	**6**
EDFA included	No	No	No	Yes	Yes	Yes
$P_{\mathrm{LO,meas}}$ [dBm]	7					
$P_{\mathrm{LO,ref}}$ [dBm]	7					
$P_{\mathrm{SI,meas}}$ [dBm]	7	−20	−40	5	−20	−40
$P_{\mathrm{SI,ref}}$ [dBm]	0					
N_{c}	40					
N_{b}	25					
$\Delta f = B_{\mathrm{eval}}$ [MHz]	500	500	50	500	500	50
$\omega_{\mathrm{SI,r}}/(2\pi)$ [GHz]	50					
$\mathcal{R}$ [A W^{-1}]	0.5					
σ [µm]	0.19	1.51	4.74	0.26	2.80	8.82
σ_{exp} [µm]	1.85	4.03	16.2	3.52	4.22	14.5

In addition to the theoretically achievable precision σ, Table S1 also specifies the experimentally demonstrated counterpart σ_{exp}. For high optical return powers, we observe a difference between the achievable and the experimentally observed standard deviation of about one order of magnitude, which reduces to a factor of $1.5-3.5$ at low powers. At high signal powers, we attribute this to our ADC, which have a rather small $\mathrm{ENOB} \approx 5.0$ in the frequency range between 10 GHz and 20 GHz, see Supplementary Section 'Noise impairments of recorded signals' above. As a consequence, the signal impairments are dominated by ADC noise rather than by shot noise, as assumed for the theoretical analysis of Table S1. The observed degradation of the measurement accuracy of approximately a factor of 10 is consistent with the fact that, for a received signal power level of 7 dBm, the ADC noise is approximately 20 dB stronger than the shot noise, see Fig. S4a. At lower received signal powers, this effect becomes less pronounced and thus the difference between the observed and the estimated distance precision decreases.

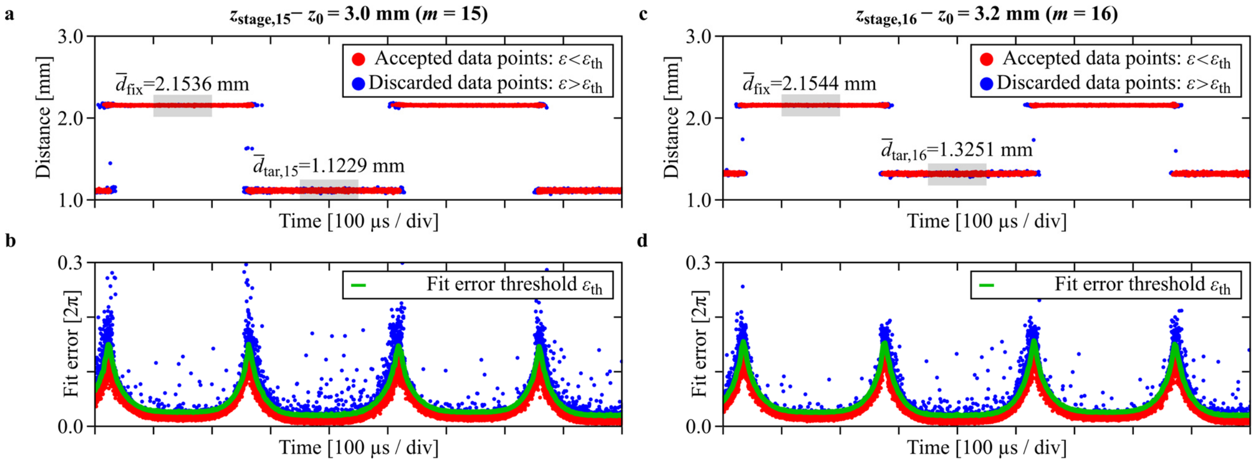

Figure S5: Evaluation of distance sweep with fiber drift compensation for two positions $z_{\text{stage},15} - z_0 = 3.0\,\text{mm}$ $(m = 15)$ and $z_{\text{stage},16} - z_0 = 3.2\,\text{mm}$ $(m = 16)$ of the target mirror. Fiber drift is eliminated by periodically comparing the variable distance d_{tar} to the target with the length d_{fix} of a fixed free-space reference beam, see Fig. S1 for details of the setup. The measurement rate amounts to $49.7\,\text{MHz}$. Data points are accepted (•, red) or discarded (•, blue) based on the respective fit error, see Supplementary Section 'Selection of reliable distance data by fit error' as well as Eq. (5) and the subsequent text in the main manuscript. **a,c** Measured distances for target mirror in position $z_{\text{stage},15} - z_0 = 3.0\,\text{mm}$ and $z_{\text{stage},16} - z_0 = 3.2\,\text{mm}$. Grey boxes mark the data points from which the average distances $\bar{d}_{\text{tar},m}$ and $\bar{d}_{\text{fix}}$ are calculated. **b,d** Fit error $\varepsilon(d_i)$ (red and blue) and fit-error threshold $\varepsilon_{\text{th}}(d_i)$ (green) of the measurements shown in Fig. S5a and Fig. S5c, respectively. Fit errors are calculated according to Eq. (5) of the main manuscript and fit-error thresholds are calculated according to Eqs. (S1) and (S2). The fit error increases in the transition regions where the chopper wheel partially blocks both beams. Between the two measurements, the target mirror is moved by 200 µm. This is well reproduced by the measured increment of 201.4 µm, obtained after accounting for a fiber drift of approximately 0.8 µm.

Evaluation of unambiguity-distance sweep with fiber drift compensation

The unambiguity-distance sweep considered in Section 'System precision and accuracy' of the main manuscript contains 16 distances that were consecutively measured over several minutes, where the time needed for each position was dictated by the data transfer from the temporal memory of our oscilloscope (ADC) to a storage network drive. During this time, the fiber lengths within our setup drift due to temperature fluctuations and mechanical vibrations, which leads to altered path lengths towards the target mirror extracted from each data set. To eliminate the effects of fiber drift, we rely on the referencing scheme described in Fig. S1, where a chopper wheel (CPW) is used to continuously switch between measuring the path length to the target mirror (red beam path) and the length of static reference beam path (grey). We set the CPW to a chopping frequency of 2 kHz, which allows to retrieve the distance to the target and the length of the reference beam path with a temporal separation of $\sim 250\,\mu\text{s}$. A subtraction of the two path length values eliminates the impact of slow fiber drifts, which typically become relevant only over a time scale of several milliseconds. In Fig. S5, we show two distance profiles obtained for two positions and $m = 16$ of the target mirror, which are separated by $200\,\mu\text{m}$. Figure S5a shows the distance profile of the first measurement ($m = 15$) for an evaluation period of $B_{\text{eval}} = 1/(10T_{\text{r}})$. Accepted data points are indicated in red, whereas discarded data points are shown in blue – for details of the selection procedure see Supplementary Section 'Selection of reliable distance data by fit error' as well as Eq. (5) and the subsequent in the main manuscript. Two alternating levels are visible, with short transition times during which the chopper wheel partially blocks both beams such that the measurement becomes unreliable. The level at $\bar{d}_{\text{fix}} = 2.1536\,\text{mm}$ corresponds to the static reference beam path, whereas the level at $\bar{d}_{\text{tar},15} = 1.1229\,\text{mm}$ represents the beam path to the target mirror. Figure S5b shows the fit error $\varepsilon(d_i)$ as well as the fit-error threshold $\varepsilon_{\text{th}}(d_i)$ for the various measurements, which exhibit a peak during the transition periods between the levels. Note that the evaluation of the fit errors allows for selection of reliable measurements even for partially blocked beams, emphasizing once more the robustness of our technique.

For further evaluation, we consider an equal number N_{accept} of accepted distance data points on each level in a temporal range of approximately 100 µs, indicated by grey boxes. We compute the point-wise difference of these data points between the upper and the lower level. The mean value of these differences then gives the path-length difference between the grey and the red beam, reduced by an integer multiple of the unambiguity distance. For the unambiguity-distance sweep, we move the target mirror by increments of 200 µm away from the beam splitter BS2 in Fig. S1 and thereby increase the length of the associated free-space beam path. The resulting distance profile for mirror position $m = 16$ is shown in Fig. S5c. In this measurement, the measured beam-path length to the target mirror has increased to $\bar{d}_{\text{tar},16} = 1.3251\,\text{mm}$, whereas the measured length of the static

beam path has changed only slightly by approximately $0.8\,\mu m$ due to fiber drift. Computing and averaging again the point-wise differences between the measured distances shown in Fig. 8c leads to an increase of the path-length difference of 201.4µm, which is in good agreement with the set increment of the mirror position.

One-port ranging system and triggering data acquisition of projectile measurements

For the high-speed ranging experiments described in Section 'High-speed ranging' of the main manuscript, we relied on the setup depicted in Fig. S6. In this setup, the optical in- and output are combined in a single output/input collimator (COL), and a fiber-optic circulator (CIRC) is used to separate the incoming receive signal from the outgoing transmit signal. Note that scheme is only useful if spurious back-reflections of the transmit signal along the path from the circulator to the target can be neglected – otherwise cyclic errors will occur and a two-port systems as shown in Fig. 2 of the main manuscript should be used. The SI comb is split at a 90/10 coupler, and the major part is fed to the circulator and is then guided to the collimator. After scattering at the target, a small portion of the SI comb enters the system through the collimator and is then guided towards the balanced photodetector by the circulator. All other components in this system are the same as for the two-port ranging system, see Fig. 2 of the main manuscript and Supplementary Section 'Detailed description of experimental setups' below for details. The collimator (Thorlabs F280APC-1550, focal length $f = 18.75\,\text{mm}$) is coated with an anti-reflective layer designed for the wavelength range of the SI comb such that the spurious back-coupling into the attached single-mode fiber can be neglected with respect to the signal returning from the target.

We used the one-port ranging system to measure surface profiles of flying air-gun projectiles, see Section 'High-speed ranging' of the main manuscript. In this experiment, we set our oscilloscope to continuously record data and to temporarily store the acquired waveforms in the internal memory. When a projectile passes the measurement beam, a part of the light is reflected and re-enters the ranging system. This is visible in the recorded data as a sharp increase of the amplitude of the recorded signal, which we use as a trigger event. Once triggered, the oscilloscope will keep the last 50 µs of data in its memory and acquire an additional 50 µs of data after the trigger event. Given the length of the projectile of approximately 7 mm and its speed of ~150 m/s, the projectile will be sampled over a period of ~50 µs. We can hence be sure that the whole projectile has been captured, independent of the temporal position of the trigger event within the acquired back-reflection signal of the projectile.

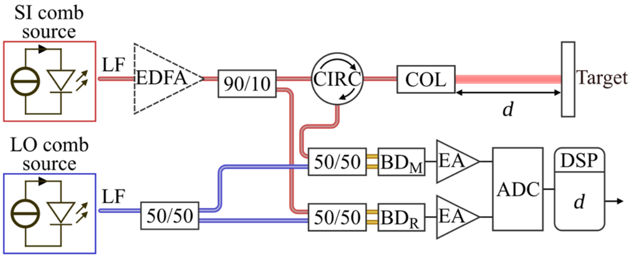

Figure S6: One-port ranging system for low-loss measurements. CIRC: Circulator; COL: output/input collimator. All other components are the same as the ones used in the two-port ranging system shown in Fig. 2 of the main manuscript. A more detailed description of the components used in the measurement system can be found in Supplementary Section 'Detailed description of experimental setups' below.

Detailed description of experimental setups

In the following, we provide a full representation of our experimental setups, see Fig. S7. Commercial model numbers of specific components are given in the caption of Fig. S7.

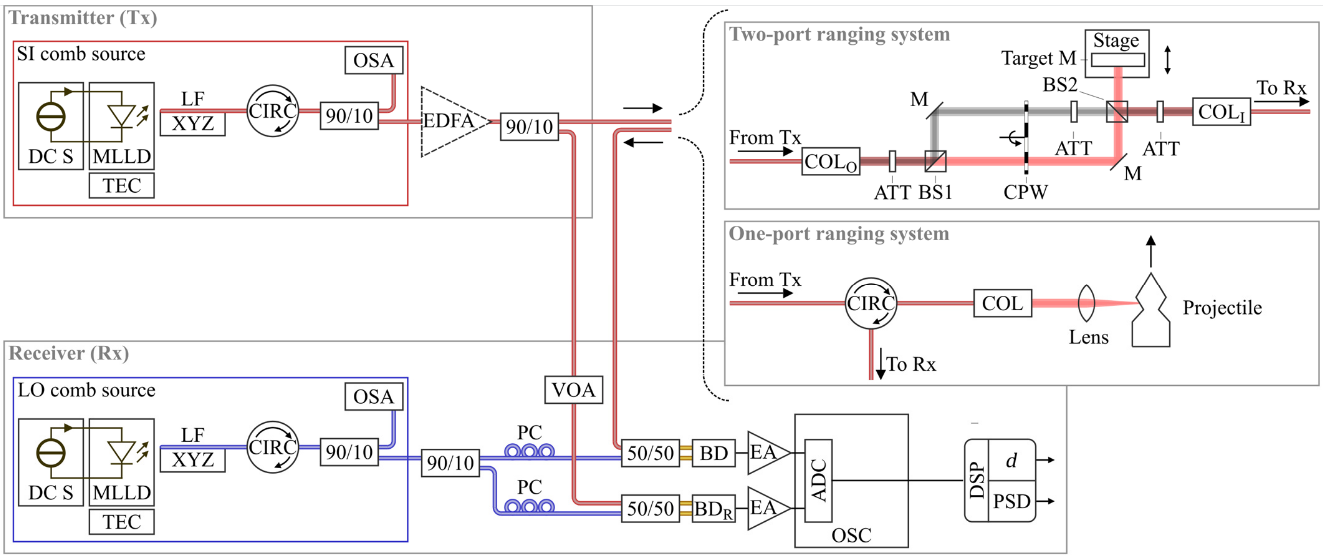

Figure S7: Full representation of the experimental setups used in our work. Optical fibers, indicated as thick red and blue lines, are standard single-mode fibers, unless specified otherwise. Components used in multiple instances with the same model number are mentioned once. **Transmitter (Tx):** DC Power supply (DC S): *ILX Lightwave LDC-3742, Newport Laser Diode Controller 6000*. Quantum-dash mode-locked laser diode (MLLD): *Custom device provided by Centre de Nanosciences et de Nanotechnologies CNRS, Université Paris-Saclay, Palaiseau, France*. Temperature controller (TEC): *Newport ILX Lightwave LTD-5910 / LTD-5910B*. Lensed fiber (LF): *OZ Optics TSMJ-3A-1550-9/125-1-7-2-12-0.9-AR*. XYZ stage (XYZ): *Thorlabs MAX313D*. Fiber-optic circulator (CIRC): Thorlabs *6015-3-APC*. Fiber-optic 90/10 coupler (90/10): *Thorlabs TN1550R2A2*. Optical spectrum analyzer (OSA): *Ando AQ6317B*. Erbium-doped fiber amplifier (EDFA): *Manlight ML-YEDFA-B-FG-33C33*. **Two-port ranging system:** Collimator (COL): *Thorlabs F280APC-1550*. Variable attenuator wheels (ATT): *Thorlabs NDC-50C-2, NDC-50C-2M*, partially in combination with neutral density filters. Beam splitters (BS): E.g., *Thorlabs BS018*. Silver mirror (M): E.g., *Thorlabs PF10-03-P01*. Chopper wheel (CPW): *SRS SR540*. Nano-precision heavy-duty stage (Stage): *Physik Instrumente M511.HD*. **One-port ranging system** High-NA lens with focal length of ~20 mm (Lens): E.g., *Thorlabs LB4854-C*. **Receiver (Rx):** Polarization controller (PC): E.g., *Thorlabs FPC032*. Variable optical attenuator (VOA): E.g., *Thorlabs VOA50-APC*. 50/50: Fiber-optic 50/50 coupler (50/50): *Thorlabs TN1550R5A2*. Balanced photodetector (BD): *Finisar BPDV2150R*. Electrical amplifier (EA): *SHF 807*. Oscilloscope (OSC): *Keysight UXR0804A*.

Supplementary references

1. Trocha, P. *et al.* Ultrafast optical ranging using microresonator soliton frequency combs. *Science.* **359**, 887–891 (2018).
2. SHF, Datasheet SHF S807-C Linear Broadband Amplifier, https://www.shf-communication.com/wp-content/uploads/datasheets/datasheet_shf_s807_c.pdf, 17.10.2021.
3. Ronnau, J., Haimov, S. & Gogineni, S. P. The effect of signal-to-noise ratio on phase measurements with polarimetric radars. *Remote Sens. Rev.* **9**, 27–37 (1994).
